# Supplementary material for: Identification of Freezing-Responsive microRNAs and Their Targets in Chinese Jujube by Small RNA and Degradome Sequencing
Source: Int J Mol Sci. 2026 Sep 4;27(17):7912. doi: 10.3390/ijms27177912 (PMC13566890; doi:10.3390/ijms27177912)
Supplement: Supplementary file 1 [file ijms-27-07912-s001.zip › Supplementary Material S2/novel_miR_42.pdf]

Provisional ID	: novel_miR_42
Score total	: 205993.6
Score for star read(s)	: 3.9
Score for read counts	: 205987.3
Score for mfe	: 0.8
Score for randfold	: 1.6
Score for cons. seed	:
Total read count	: 404047
Mature read count	: 396583
Loop read count	: 14
Star read count	: 7450

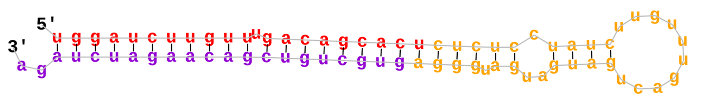

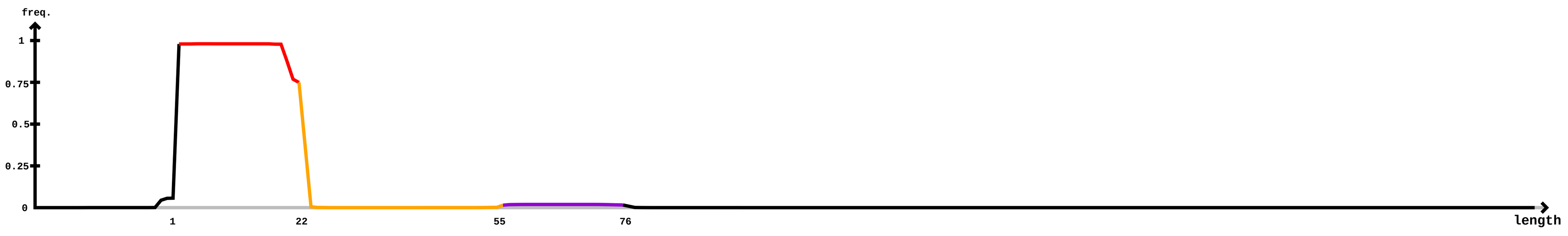

Mature Star

```
5'- uccaucuuguucguuuacccucuggaucuuguuugacagcacucucuccuaucuuguuugacugaugaugaugggagugcugucgacaagaucuagaagguacugucgaauaagauacagauacggaggaaggaucaacuuuaccuacucucacacaaucuucuucauauucucuucaauuuuauuuuuuuauuguucguauuuuuuucccagauuagauuucccuguuaaucaccaugguauuaaca -3'  obs
    uccaucuuguucguuuacccucuggaucuuguuugacagcacucucuccuaucuuguuugacugaugaugaugggagugcugucgacaagaucuagaagguacugucgaauaagauacagauacggaggaaggaucaacuuuaccuacucucacacaaucuucuucauauucucuucaauuuuauuuuuuuauuguucguauuuuuuucccagauuagauuucccuguuaaucaccaugguauuaaca       exp
    ...((((((((((..((((.(((((((((((((.(((((((((((((.((((.........))))..)).))))))))))))))))))))))))).))))....))))))))))..(((((((((((((.........................)))))))..........((((............))))..)))))))......................(((((((.((....)))))))))   reads  mm  sample
    .ccaucuuguucguuuacc.......................................   1   0   LT-1
    .ccaucuuguucguuuacccucugg.................................   5   0   LCK-3
    .ccaucuuguucguuuacccucuggauc..............................   1   0   LT-3
    ...aucuuguucguuuacccucugga................................   2   0   JCK-1
    .....cuuguucguuuacccucuggauc..............................   4   0   LT-2
    ......uuguucguuuaccUucuggauc..............................   2   1   JCK-1
    ......uuguucguuuaccUucuggauc..............................   2   1   LT-2
    ......uuguucguuuaccUucuggauc..............................   6   1   JCK-2
    ......uuguucguuuaccUucuggauc..............................   3   1   JCK-3
    .......uguucguuuacccucuggauc..............................   1   0   JCK-2
    .......uguucguuuaccUucuggauc..............................   12  1   LT-3
    .......uguucguuuaccUucuggauc..............................   30  1   JCK-3
    .......uguucguuuaccUucuggauc..............................   3   1   LT-2
    .......uguucguuuacccucuggauc..............................   2   0   JCK-3
    .......uguucguuuacccucuggauc..............................   1   0   JCK-1
    .......uguucguuuaccUucuggauc..............................   12  1   LT-1
    .......uguucguuuaccUucuggauc..............................   10  1   JCK-1
    .......uguucguuuaccUucuggauc..............................   31  1   JCK-2
    ..........ucguuuacccucuggaucuug...........................   1   0   LT-2
    ............guuuacccucuggaucuuguuugaca....................   2   0   LCK-3
    .............uuuacccucuggaucuuguA.........................   1   1   JCK-1
    .............uuuacccucuggaucuuguuu........................   2   0   JCK-3
    .............uuuacccucuggaucuuguuuga......................   1   0   JCK-1
    ...............uacccucuggaucuuguuugacagc..................   2   0   JCK-2
    ................Ccccucuggaucuuguuuga......................   1   1   LT-1
    ................acccucuggaucuuguuugacagc..................   2   0   JCK-2
    ................acccucuggaucuuguuugacagca.................   2   0   JCK-3
    ................acccucuggaucuuguuugacagca.................   2   0   JCK-2
    .................cccucuggaucuuguuugacagc..................   1   0   LT-3
    ..................ccucuggaucuuguuugac.....................   3   0   JCK-2
    ..................ccucuggaucuuguuugaca....................   2   0   JCK-1
    ..................ccucuggaucuuguuugaca....................   2   0   JCK-3
    ..................ccucuggaucuuguuugacag...................   1   0   JCK-3
```

Mature **Star**

uccaucuuguucguuuacccucuggaucuuguuugacagcacucucuccuaucuuguuugacugaugaugaugggagugcugucgacaagaucuagaagguacugucgaauaagauacagauacggaggaaggaucaacuuuaccuacucucacacaaucuucuucauauucucuucaauuuuuauuuuuuuauuguucguauuuuuuuucccagauuagauuucccuguuaaucaccaugguauuaaca			
..................ccucuggaucuuguuugacag	4	0	LCK-3
..................ccucuggaucuuguuugacag	1	0	JCK-1
..................ccucuggaucuuguuugacag	1	0	JCK-2
..................ccucuggaucuuguuugacagc	35	0	JCK-3
..................ccucuggaucuuguuugacagc	21	0	JCK-1
..................ccucuggaucuuguuugacagc	9	0	LT-3
..................ccucuggaucuuguuugacagc	6	0	LT-1
..................ccucuggaucuuguuugacagc	32	0	JCK-2
..................ccucuggaucuuguuugacagc	3	0	LT-2
..................ccucuggaucuuguuugacagc	6	0	LCK-3
..................ccucuggaucuuguuugacagcU	1	1	JCK-3
..................ccucuggaucuuguuugacagcU	2	1	JCK-1
..................ccucuggaucuuguuugacagcU	4	1	JCK-2
...................cucuggaucuuguuugac	13	0	LT-2
...................cucuggaucuuguuugaU	2	1	JCK-3
...................cucuggCucuuguuugac	1	1	JCK-2
...................cucuggaucuuguuugac	10	0	JCK-3
...................cucuggaucuuguuugac	13	0	LT-3
...................cucuggaucuuguuugac	6	0	LCK-3
...................cucuggaucuuguuugac	13	0	JCK-2
...................cucuggaucuuguuugac	8	0	LT-1
...................cucuggaucuuguuugac	4	0	JCK-1
...................cucuggCucuuguuugaca	1	1	JT-1
...................cucuggaucuuguuugaca	21	0	JT-3
...................cucuggaucuuguuugaUa	1	1	JCK-2
...................cucuggaucuuguuugacC	1	1	LT-1
...................cucuggCucuuguuugaca	1	1	LCK-1
...................cucuggaucuuguuugaca	78	0	LCK-3
...................cucuggaucuuguuugaca	65	0	LT-3
...................cucuggaucuuguuugaca	27	0	LT-1
...................cucuggaucuuguuugacU	1	1	LT-3
...................cucuggaucuuguuugaca	42	0	LCK-1
...................cucuggaucuuguuugacU	14	1	LCK-3
...................cucuggaucuuguuugaca	18	0	JCK-3
...................cucuggaucuuguuugacU	7	1	JCK-2
...................cucuggaucuuguuugacU	6	1	JCK-3
...................cucuggaucuuguuugaca	41	0	JCK-2
...................cucuggaucuuguuugaca	18	0	JT-2
...................cuNuggaucuuguuugaca	1	1	LT-2
...................cucuggaucuuguuugacU	6	1	LT-2
...................cucuggaucuuguuugacU	2	1	LT-1
...................cucuggaucuuguuugaca	29	0	LCK-2
...................cucuggaucuuguuugaca	62	0	LT-2
...................cucuggaucuuguuugaca	28	0	JT-1
...................cucuggaucuuguuugaca	20	0	JCK-1
...................cucuggaucuuguuugaUa	1	1	JCK-3
...................cucuggaucuuAuuugacag	1	1	LT-2
...................cucuggaucuuguuugacag	15	0	JT-3
...................cucuggaucuuguuugacag	7	0	LT-3
...................cucuggaucuuguuugacag	5	0	JCK-1
...................cucuggaucuuguuugacag	12	0	LT-2
...................cucuggaucuuguuugacag	4	0	JCK-2
...................cucuggaucuuguuugacaU	3	1	LT-3
...................cucuggaucuuguuugacag	23	0	LCK-3
...................cucuggaucuuguuugacag	6	0	JCK-3
...................cucuggaucuuguuugacaU	2	1	LT-1
...................cucuggaucuuguuugacag	7	0	LT-1
...................cucuggaucuuguuugacag	14	0	JT-2
...................cucuggaucuuAuuugacagc	1	1	LT-3
...................cucuggaucuuguuugacagc	1379	0	LCK-3
...................cucCggaucuuguuugacagc	1	1	LT-2
...................cucuggaucuuguuugaUagc	1	1	LT-3
...................cucuggaucuuAuuugacagc	2	1	JCK-2
...................cucuggaucuuguuugacCgc	2	1	JCK-2
...................cucuggaucuuguuugacagU	2	1	LT-3
...................cucuggaucuuguuugCcagc	5	1	LT-3
...................cucuggaucuuguCugacagc	1	1	JCK-3
...................cucuggaucuCguuugacagc	1	1	LCK-3
...................cucuggaucuuguuugCcagc	2	1	LCK-3
...................cucuggaucuuguuugCcagc	7	1	LT-2

```
                     Mature                                                Star
uccaucuuguucguuuacccucuggaucuuguuugacagcacucucuccuaucuuguuugacugaugaugaugggagugcugucgacaagaucuagaagguacugucgaauaagauacagauacggaggaaggaucaacuuuaccuacucucacacaaucuucuucauauucucuucaauuuuuauuuuuuuauuguucguauuuuuuuucccagauuagauuucccuguuaaucaccaugguauuaaca
...................cucuUgaucuuguuugacagc..................................................................................................................................................................................................................  1     1   LT-3
...................cucuggaucuuguuugacGgc..................................................................................................................................................................................................................  1     1   LT-3
...................cucuggaucuuguuugacagU..................................................................................................................................................................................................................  1     1   JCK-3
...................cucuggaucuuguuugaUagc..................................................................................................................................................................................................................  2     1   JCK-3
...................cucuAgaucuuguuugacagc..................................................................................................................................................................................................................  1     1   JCK-1
...................cuUuggaucuuguuugacagc..................................................................................................................................................................................................................  3     1   JCK-3
...................cucuggauAuuguuugacagc..................................................................................................................................................................................................................  1     1   LT-1
...................cucuggaucuuguuugacagc..................................................................................................................................................................................................................  2907  0   JCK-2
...................cucuggaucuuguuugacagc..................................................................................................................................................................................................................  90    0   JT-3
...................cCcuggaucuuguuugacagc..................................................................................................................................................................................................................  1     1   JCK-2
...................cNcuggaucuuguuugacagc..................................................................................................................................................................................................................  1     1   LCK-1
...................cNcuggaucuuguuugacagc..................................................................................................................................................................................................................  4     1   JCK-2
...................cucuAgaucuuguuugacagc..................................................................................................................................................................................................................  1     1   JCK-2
...................cCcuggaucuuguuugacagc..................................................................................................................................................................................................................  1     1   LCK-3
...................cucCggaucuuguuugacagc..................................................................................................................................................................................................................  2     1   JCK-2
...................cCcuggaucuuguuugacagc..................................................................................................................................................................................................................  1     1   LCK-2
...................cNcuggaucuuguuugacagc..................................................................................................................................................................................................................  1     1   LT-3
...................cucuggaucuuguuugCcagc..................................................................................................................................................................................................................  9     1   LT-1
...................cucuggaucuuguuugacaNc..................................................................................................................................................................................................................  4     1   JCK-1
...................cucNggaucuuguuugacagc..................................................................................................................................................................................................................  2     1   JCK-1
...................cucuggaucuuguuugaGagc..................................................................................................................................................................................................................  1     1   JCK-1
...................cucuggaucuuguuugacagc..................................................................................................................................................................................................................  2088  0   JCK-3
...................cucuggaucuuguuugCcagc..................................................................................................................................................................................................................  4     1   JCK-1
...................cucuggaucuuguuugacagc..................................................................................................................................................................................................................  62    0   JT-2
...................cucuggaucuuguuugacCgc..................................................................................................................................................................................................................  2     1   LT-1
...................cucuggaucuuguuugCcagc..................................................................................................................................................................................................................  1     1   JCK-3
...................cucuggaucuuguuugacCgc..................................................................................................................................................................................................................  4     1   LT-3
...................cucuggaucuuguuugacCgc..................................................................................................................................................................................................................  2     1   JCK-3
...................cucuggCucuuguuugacagc..................................................................................................................................................................................................................  20    1   LT-2
...................cucuggCucuuguuugacagc..................................................................................................................................................................................................................  1     1   LCK-2
...................cucuggaucuuguuugacagc..................................................................................................................................................................................................................  275   0   LCK-1
...................cuUuggaucuuguuugacagc..................................................................................................................................................................................................................  2     1   JCK-1
...................cucuggCucuuguuugacagc..................................................................................................................................................................................................................  4     1   JCK-3
...................cucCggaucuuguuugacagc..................................................................................................................................................................................................................  3     1   LT-3
...................cucuggaucuuguuugacCgc..................................................................................................................................................................................................................  2     1   LT-2
...................cucuggaucuuguuugacagA..................................................................................................................................................................................................................  3     1   LT-2
...................cucuggaucuuguuugGcagc..................................................................................................................................................................................................................  1     1   JCK-3
...................cuGuggaucuuguuugacagc..................................................................................................................................................................................................................  1     1   LT-2
...................cucuggaucuuguuugacagc..................................................................................................................................................................................................................  1675  0   LT-2
...................cucuggCucuuguuugacagc..................................................................................................................................................................................................................  4     1   LCK-3
...................cucuggaucuuguCugacagc..................................................................................................................................................................................................................  1     1   JCK-2
...................cucuggaucuuguuugaUagc..................................................................................................................................................................................................................  2     1   JCK-2
...................cucuggaucuuguuugacagc..................................................................................................................................................................................................................  292   0   LCK-2
...................cucuggaCcuuguuugacagc..................................................................................................................................................................................................................  2     1   LT-1
...................cNcuggaucuuguuugacagc..................................................................................................................................................................................................................  1     1   JCK-3
...................cucuggaucuuguuugacagc..................................................................................................................................................................................................................  3001  0   LT-3
...................cCcuggaucuuguuugacagc..................................................................................................................................................................................................................  2     1   LT-2
...................cucugAaucuuguuugacagc..................................................................................................................................................................................................................  1     1   JCK-3
...................cucuggaucuuguuugacCgc..................................................................................................................................................................................................................  2     1   LCK-3
...................cucGggaucuuguuugacagc..................................................................................................................................................................................................................  1     1   JCK-1
...................cucuggaucuuUuuugacagc..................................................................................................................................................................................................................  1     1   JCK-3
...................cucugNaucuuguuugacagc..................................................................................................................................................................................................................  1     1   LCK-1
...................cucuggaucuuguuugacagc..................................................................................................................................................................................................................  66    0   JT-1
...................cucuggaucuuguuuUacagc..................................................................................................................................................................................................................  1     1   JCK-2
...................cucuUgaucuuguuugacagc..................................................................................................................................................................................................................  1     1   LT-2
...................cucuAgaucuuguuugacagc..................................................................................................................................................................................................................  4     1   LT-2
...................cucuggaucuuNuuugacagc..................................................................................................................................................................................................................  3     1   JCK-1
...................cucuggaucuuguuugacCgc..................................................................................................................................................................................................................  2     1   JCK-1
...................cucuggaucuCguuugacagc..................................................................................................................................................................................................................  2     1   LT-2
...................cucuggaucuuguuuAacagc..................................................................................................................................................................................................................  1     1   LCK-3
...................cucuggaucuuguuugacagc..................................................................................................................................................................................................................  1680  0   JCK-1
...................cucuggauUuuguuugacagc..................................................................................................................................................................................................................  1     1   LT-1
...................cucCggaucuuguuugacagc..................................................................................................................................................................................................................  2     1   JCK-3
...................cucuggaucuCguuugacagc..................................................................................................................................................................................................................  1     1   LT-1
...................cuAuggaucuuguuugacagc..................................................................................................................................................................................................................  2     1   JCK-3
...................cucuggauNuuguuugacagc..................................................................................................................................................................................................................  2     1   JCK-3
...................cucuggaucuuUuuugacagc..................................................................................................................................................................................................................  1     1   LT-1
...................cucuggCucuuguuugacagc..................................................................................................................................................................................................................  11    1   LT-1
...................cucuAgaucuuguuugacagc..................................................................................................................................................................................................................  1     1   LCK-3
...................cucuggCucuuguuugacagc..................................................................................................................................................................................................................  2     1   LCK-1
```

```
                      Mature                                                Star
uccaucuuguucguuuacccucuggaucuuguuugacagcacucucuccuaucuuguuugacugaugaugaugggagugcugucgacaagaucuagaagguacugucgaauaagauacagauacggaggaaggaucaacuuuaccuacucucacacaaucuucuucauauucucuucaauuuuuauuuuuuuauuguucguauuuuuuuucccagauuagauuucccuguuaaucaccaugguauuaaca
...................cuNuggaucuuguuugacagc..........................................................................................................................................................................................  2     1  LT-2
...................cCcuggaucuuguuugacagc..........................................................................................................................................................................................  1     1  LT-1
...................cucuggaucuuguuuAacagc..........................................................................................................................................................................................  1     1  LT-1
...................cucuggaucuuguuugCcagc..........................................................................................................................................................................................  3     1  JCK-2
...................cucuggaucCuguuugacagc..........................................................................................................................................................................................  1     1  LT-1
...................cucCggaucuuguuugacagc..........................................................................................................................................................................................  1     1  LT-1
...................cucuggaucuuAuuugacagc..........................................................................................................................................................................................  1     1  LT-1
...................cucuAgaucuuguuugacagc..........................................................................................................................................................................................  3     1  LT-3
...................cucuggCucuuguuugacagc..........................................................................................................................................................................................  1     1  JCK-1
...................cucugAaucuuguuugacagc..........................................................................................................................................................................................  1     1  LT-3
...................cucuggaucCuguuugacagc..........................................................................................................................................................................................  1     1  JCK-2
...................cucuggaucuuguuugacagc..........................................................................................................................................................................................  3319  0  LT-1
...................cucugUaucuuguuugacagc..........................................................................................................................................................................................  1     1  LT-1
...................cucuggCucuuguuugacagc..........................................................................................................................................................................................  11    1  JCK-2
...................cucuggCucuuguuugacagc..........................................................................................................................................................................................  11    1  LT-3
...................cGcuggaucuuguuugacagc..........................................................................................................................................................................................  1     1  LT-2
...................cNcuggaucuuguuugacagc..........................................................................................................................................................................................  2     1  JCK-1
...................cucuggaucuugCuugacagc..........................................................................................................................................................................................  1     1  JCK-2
...................cucugAaucuuguuugacagc..........................................................................................................................................................................................  3     1  LT-2
...................cucuggaucuuguuugacagcC.........................................................................................................................................................................................  2     1  LT-1
...................cucuggaucuuguuugacagca.........................................................................................................................................................................................  27    0  LT-1
...................cucuggaucuugCuugacagca.........................................................................................................................................................................................  1     1  JCK-2
...................cucuggaucuuguuugacagca.........................................................................................................................................................................................  87    0  JCK-3
...................cucuggaucuuguuugacagca.........................................................................................................................................................................................  22    0  LT-3
...................cucuggaucuuguuugacagcU.........................................................................................................................................................................................  13    1  JT-2
...................cucuggaucuuguuugacagcC.........................................................................................................................................................................................  2     1  LT-2
...................cucuggaucuuguuuNacagca.........................................................................................................................................................................................  1     1  JCK-3
...................cucuggaucuuguuugacagcG.........................................................................................................................................................................................  1     1  JT-2
...................cucuggaucuuguuugacagca.........................................................................................................................................................................................  5     0  LCK-3
...................cucuggaucuuguuugacagcC.........................................................................................................................................................................................  1     1  JCK-2
...................cucuggaucuuguuugacagca.........................................................................................................................................................................................  19    0  LT-2
...................cucuggaucuuguuugacagca.........................................................................................................................................................................................  117   0  JCK-2
...................cucuggaucuuguuugacagca.........................................................................................................................................................................................  1     0  JT-2
...................cucuggaucuuguuugacagcU.........................................................................................................................................................................................  66    1  LT-1
...................cucuggaucuuguuugacagcU.........................................................................................................................................................................................  47    1  LT-2
...................cucuggaucuuguuugacagca.........................................................................................................................................................................................  72    0  JCK-1
...................cucuggaucuuguuugacagcU.........................................................................................................................................................................................  29    1  JCK-3
...................cucuggaucuuguuugacagcC.........................................................................................................................................................................................  2     1  LT-3
...................cucuggaucuuguuugacagcU.........................................................................................................................................................................................  55    1  JCK-2
...................cucuggaucuuguuugacagcU.........................................................................................................................................................................................  84    1  LCK-3
...................cucuggaucuuguuugacagcU.........................................................................................................................................................................................  69    1  LT-3
...................cucuggaucuuguuugacagcU.........................................................................................................................................................................................  25    1  JCK-1
...................cucuggaucuuguuugacagcU.........................................................................................................................................................................................  14    1  JT-3
...................cucuggaucuuguuugacagca.........................................................................................................................................................................................  10    0  JT-3
...................cucuggaucuuguuugacagcac........................................................................................................................................................................................  1     0  LT-1
...................cucuggaucuuguuugacagcac........................................................................................................................................................................................  1     0  LT-3
...................cucuggaucuuguuugacagcacN.......................................................................................................................................................................................  1     1  LT-2
...................cucuggaucuuguuugacagcacu.......................................................................................................................................................................................  1     0  LT-2
....................ucuggaucuuguuugaca............................................................................................................................................................................................  3     0  LCK-2
....................ucuggaucuuguuugaca............................................................................................................................................................................................  28    0  LT-1
....................ucuggaucuuguuugaca............................................................................................................................................................................................  33    0  JCK-2
....................ucuggaucuuguuugaca............................................................................................................................................................................................  15    0  JCK-3
....................ucuggaucuuguuugaca............................................................................................................................................................................................  35    0  JT-3
....................ucuggaucuuguuugaca............................................................................................................................................................................................  41    0  LT-2
....................ucuggaucuuguuugacU............................................................................................................................................................................................  6     1  LT-1
....................ucuggaucuuguuugacU............................................................................................................................................................................................  2     1  LT-3
....................ucuggaucuuguuugaca............................................................................................................................................................................................  25    0  JCK-1
....................ucuggaucuuguuugaca............................................................................................................................................................................................  79    0  LCK-3
....................ucuggaucuuguuugaca............................................................................................................................................................................................  9     0  JT-1
....................ucuggaucuuguuugaca............................................................................................................................................................................................  26    0  LT-3
....................ucuggaucuuguuugacU............................................................................................................................................................................................  1     1  JCK-1
....................ucuggaucuuguuugacU............................................................................................................................................................................................  9     1  LCK-3
....................ucuggaucuuguuugaca............................................................................................................................................................................................  3     0  LCK-1
....................ucuggaucuuguuugaca............................................................................................................................................................................................  30    0  JT-2
....................ucuggaucuuguuugacU............................................................................................................................................................................................  1     1  JCK-2
....................ucuggaucuuguuugacag...........................................................................................................................................................................................  2     0  LT-1
....................ucuggaucuuguuugacagc..........................................................................................................................................................................................  63    0  LCK-1
....................ucuggaucuuguuugacagc..........................................................................................................................................................................................  107   0  LT-1
....................ucuggaucuuguuugacagc..........................................................................................................................................................................................  29    0  JCK-1
....................ucuggaucuuguuugacagA..........................................................................................................................................................................................  1     1  LT-3
```

Mature Star

uccaucuuguucguuuacccucuggaucuuguuugacagcacucucuccuaucuuguuugacugaugaugaugggagugcugucgacaagaucuagaagguacugucgaauaagauacagauacggaggaaggaucaacuuuaccuacucucacacaaucuucuucauauucucuucaauuuuuauuuuuuuauuguucguauuuuuuuucccagauuagauuucccuguuaaucaccaugguauuaaca			
....................ucuggaucuuguuugacagc	127	0	LT-3
....................ucuggaucuuguuugacagc	35	0	JCK-3
....................ucuggaucuuguuugacagc	116	0	LT-2
....................ucuggaucuuguuugacagA	1	1	JCK-3
....................ucuggaucuuguuugacaUc	1	1	LT-1
....................ucuggCucuuguuugacagc	1	1	LCK-1
....................ucuggaucuuguuugacagc	41	0	LCK-3
....................ucuggaucuuguuuNacagc	1	1	LCK-1
....................ucuggaucuuguuugacagc	75	0	LCK-2
....................uGuggaucuuguuugacagc	1	1	LCK-1
....................ucuggaucuuguuugacagc	55	0	JCK-2
....................ucuggCucuuguuugacagc	2	1	LT-2
....................ucuggaucuuguuugacagA	1	1	JCK-1
....................ucuggaucuuguuuUacagc	1	1	LCK-2
....................uUuggaucuuguuugacagca	1	1	JCK-1
....................ucuggaucCuguuugacagca	1	1	JCK-2
....................ucuggaucuuguuugacagcU	6	1	LT-1
....................ucuggaucuuguuugacagca	299	0	LT-1
....................ucuggaucuuguuugacagcU	3	1	JCK-2
....................ucuggaucuuguuugacagca	628	0	JCK-1
....................ucuNgaucuuguuugacagca	1	1	JCK-1
....................ucuggaucuCguuugacagca	1	1	LT-3
....................ucuggaucuuguuugacagcC	1	1	LCK-3
....................ucuggaucuuguuugacagcU	14	1	LT-2
....................ucuggaucuuguuugacagcU	6	1	JCK-1
....................ucuggaucuuguuugacagca	9	0	LCK-1
....................ucuggaucuuguuugCcagca	2	1	LT-3
....................ucuggaucuuguuugacagca	195	0	LT-2
....................ucuggaucuuguuugacaUca	2	1	JCK-1
....................uUuggaucuuguuugacagca	8	1	JCK-3
....................ucuggaucuuguuugacagcU	3	1	JCK-3
....................ucuggaucuuguuugacagcU	7	1	LT-3
....................ucuggaucuuguuugacagcN	1	1	JCK-1
....................ucuggaucuugGuugacagca	1	1	JCK-1
....................ucuggaucuuguuugacaUca	5	1	JCK-2
....................uUuggaucuuguuugacagca	12	1	JCK-2
....................ucuggaucuuguuugacagca	17	0	LCK-2
....................ucuggaucuuguuugacagca	145	0	LCK-3
....................ucCggaucuuguuugacagca	1	1	JCK-1
....................ucuggaucuuguuugUcagca	1	1	JCK-3
....................ucuggaucuuguuugGcagca	1	1	LT-2
....................ucuggaucuuguuugCcagca	1	1	JCK-1
....................ucuggaucuuguuugGcagca	1	1	LCK-3
....................ucuggaucuuguuugacagca	832	0	JCK-3
....................ucuggaucuuguuugacagcU	9	1	LCK-3
....................ucuggaucuuguuugUcagca	1	1	JCK-2
....................uNuggaucuuguuugacagca	1	1	JCK-1
....................ucuggaucuuguuugacagUa	1	1	JCK-2
....................ucuggaucuuguuugacGgca	2	1	JCK-1
....................ucuggaucuuguuugacagNa	2	1	JCK-1
....................ucuggCucuuguuugacagca	1	1	JCK-2
....................ucuggaucuugGuugacagca	2	1	LT-1
....................ucuggaucuuguuugacaUca	3	1	JCK-3
....................ucuggaucuuguuugacagca	11	0	JT-2
....................ucuggaucuuguuugacagca	1097	0	JCK-2
....................ucuggauUuuguuugacagca	1	1	JCK-2
....................ucCggaucuuguuugacagca	1	1	JCK-2
....................ucuggaucuuguuugacagca	29	0	JT-1
....................ucuggaucuuguuugacagca	12	0	JT-3
....................ucuggauAuuguuugacagca	1	1	JCK-2
....................ucuggaucuuguuugacagca	267	0	LT-3
....................ucuggaucuuguuugCcagca	1	1	LT-1
....................ucuggaucuuguuugacagcac	27	0	LT-1
....................ucuggaucuuguuugacagcaU	2	1	LT-2
....................ucuggaucuuguuugacagcac	22	0	JCK-3
....................ucuggaucuuguuugCcagcac	1	1	LT-2
....................ucuggaucuuguuugacagcac	19	0	LCK-3
....................ucuggaucuuguuugacagcac	17	0	LT-2
....................ucuggaucuuguuugacagcac	24	0	LT-3
....................ucuggaucuuguuugacagcac	50	0	JCK-2

Mature Star

uccaucuuguucguuuacccucuggaucuuguuugacagcacucucuccuaucuuguuugacugaugaugaugggagugcugucgacaagaucuagaagguacugucgaauaagauacagauacggaggaaggaucaacuuuaccuacucucacacaaucuucuucauauucucuucaauuuuuauuuuuuuauuguucguauuuuuuuucccagauuagauuucccuguuaaucaccaugguauuaaca

....................ucuggaucuuguuugacagcaU....	1	1	LT-1
....................ucuggaucuuguuugacagcac....	34	0	JCK-1
....................ucuggaucuuguuugacagcacu....	3	0	LT-1
....................ucuggaucuuguuugacagcacu....	5	0	JCK-2
....................ucuggaucuuguuugacagcaUu....	1	1	LT-3
....................ucuggaucuuguuugacagcacu....	2	0	JCK-3
....................ucuggaucuuguuugacagcacu....	2	0	JCK-1
....................ucuggaucuuguuugacagcacuc....	1	0	JCK-3
....................ucuggaucuuguuugacagcacuc....	1	0	JCK-1
....................ucuggaucuuguuugacagcacuc....	1	0	JCK-2
....................ucuggaucuuguuugacagcacuc....	2	0	LT-3
....................ucuggaucuuguuugacagcacucu....	2	0	LT-1
.....................cuggaucuuguuugacagc....	1	0	LT-3
.....................cuggaucuuguuugacagc....	5	0	LT-1
.....................cuggaucuuguuugacagc....	13	0	LT-2
.....................cuggaucuuguuugacagc....	1	0	JCK-1
.....................cuggaucuuguuugacagc....	2	0	JCK-3
.....................cuggaucuuguuugacagc....	5	0	LCK-3
.....................cuggaucuuguuugacagc....	7	0	JCK-2
.....................cuggaucuuguuugacagca....	51	0	JCK-3
.....................cuggaucuuguuugacagcU....	1	1	JCK-3
.....................cuggaucuuguuugacagca....	61	0	JCK-2
.....................cuggaucuuguuugacagca....	11	0	LT-2
.....................cuggaucuuguuugacagcU....	5	1	LT-2
.....................cuggaucuuguuugacagcU....	1	1	JCK-2
.....................cuggaucuuguuugacagca....	12	0	LCK-3
.....................cuggaucuuguuugCcagca....	1	1	JCK-1
.....................cuggaucuuguuugacagca....	58	0	JCK-1
.....................cuggaucuuguuugacagca....	15	0	LT-3
.....................cuggaucuuguuugacagca....	9	0	LT-1
.....................cuggaucuuguuugacagcU....	4	1	JT-3
.....................cuggaucuuguuugacagcU....	4	1	JT-2
.....................cuggaucuuguuugacagcU....	1	1	LT-3
.....................cuggaucuuguuugacagcac....	4	0	LT-2
.....................cuggaucuuguuugacagcac....	1	0	LCK-3
.....................cuggaucuuguuugacagcac....	3	0	LT-3
.....................cuggaucuuguuugacagcacu....	4	0	LT-1
.....................cuggaucuuguuugacagcacu....	17	0	JCK-1
.....................cuggaucuuguuugacagcaUu....	1	1	JCK-2
.....................cuggaucuuguuugacagcacu....	7	0	LT-2
.....................cuggaucuuguuugacagcacu....	46	0	JCK-2
.....................cuggaucuuguuugacagcaUu....	2	1	JCK-3
.....................cuggaucuuguuugacagcacu....	15	0	LCK-3
.....................cuggaucuuguuugacagcacu....	22	0	JCK-3
.....................cuggaucuuguuugacagcacu....	13	0	LT-3
.....................cuggaucuuguuugacagcacuc....	1	0	JCK-1
.....................cuggaucuuguuugacagcacuc....	3	0	JCK-3
.....................cuggaucuuguuugacagcacuc....	1	0	JCK-2
.....................cuggaucuuguuugacagcacucu....	3	0	JCK-2
.....................cuggaucuuguuugacagcacucuc....	1	0	JCK-1
.....................cuggaucuuguuugacagcacucuc....	3	0	JCK-2
......................uggaucuuguuuUacagc....	1	1	JT-2
......................uggCucuuguuugacagc....	3	1	JT-1
......................uggNucuuguuugacagc....	1	1	JCK-1
......................uggaucuuguuugacCgc....	1	1	JT-1
......................ugAaucuuguuugacagc....	3	1	LT-2
......................uggaucuuguuugacagU....	13	1	LCK-3
......................Cggaucuuguuugacagc....	1	1	LT-3
......................uggaucuuguuNgacagc....	1	1	JCK-1
......................uNgaucuuguuugacagc....	1	1	JCK-1
......................uUgaucuuguuugacagc....	1	1	LCK-3
......................uggaucuuguuugacagA....	7	1	LT-2
......................uAgaucuuguuugacagc....	5	1	LT-2
......................uggaucuugCuugacagc....	1	1	JCK-1
......................uggaucuuguuugaNagc....	11	1	LCK-1
......................uggaucuuguuNgacagc....	1	1	JT-1
......................ugUaucuuguuugacagc....	1	1	LCK-3
......................uNgaucuuguuugacagc....	2	1	LCK-1
......................uggaucuuguuugCcagc....	15	1	LCK-3
......................uggaucuuguGugacagc....	1	1	LT-2

Mature **Star**

uccaucuuguucguuuacccucuggaucuuguuugacagcacucucuccuaucuuguuugacugaugaugaugggga**gugcugucgacaagaucuaga**agguacugucgaauaagauacagauacggaggaaggaucaacuuuaccuacucucacacaaucuucuucauauucucuucaauuuuuauuuuuuuauuguucguauuuuuuuucccagauuagauuucccuguuaaucaccaugguauuaaca

uggaucuuguuugCcagc	2	1	LCK-2
uggaucuuguuugacagc	1810	0	JCK-2
uggaucuCguuugacagc	1	1	LT-1
uggCucuuguuugacagc	7	1	LT-3
uggGucuuguuugacagc	1	1	LCK-3
uggaucuuguuugacagc	520	0	JT-1
uggaucuugCuugacagc	1	1	JCK-2
uggCucuuguuugacagc	10	1	LCK-3
uggaucuuguuugacCgc	2	1	LCK-3
ugAaucuuguuugacagc	1	1	LT-1
uggaucuuguuugCcagc	24	1	LT-2
uggCucuuguuugacagc	1	1	JCK-3
uggaucuuguuugCcagc	1	1	JCK-3
uggaucuuguuugacagG	1	1	JCK-1
ugAaucuuguuugacagc	2	1	LCK-1
uggaucuuguuugacCgc	2	1	JCK-2
uggaucuuAuuugacagc	3	1	LT-2
uCgaucuuguuugacagc	1	1	LT-1
uggaucuuguuugacagc	353	0	JT-2
uggCucuuguuugacagc	5	1	LT-1
uggaucuuguuNgacagc	1	1	JCK-2
uggaucuuguuugacCgc	2	1	LCK-2
uggCucuuguuugacagc	3	1	LCK-2
uggaucCuguuugacagc	1	1	LT-3
uggCucuuguuugacagc	2	1	LCK-1
ugUaucuuguuugacagc	1	1	JCK-1
uggaucuuguuugacagU	12	1	LT-3
uggaucuuAuuugacagc	1	1	LCK-2
uggaucuuguuugacagc	1255	0	JCK-3
uggNucuuguuugacagc	1	1	JT-2
uggaucuugCuugacagc	1	1	LT-1
uUgaucuuguuugacagc	1	1	LT-3
uggaucuuguuugacagc	5296	0	LT-2
uggaucuuguuugacagA	4	1	LCK-3
uAgaucuuguuugacagc	4	1	LCK-3
ugUaucuuguuugacagc	1	1	LT-2
uggaucuuguuugCcagc	6	1	LT-1
uggaucuAguuugacagc	1	1	LT-1
uggaucuuguuugacagU	1	1	JCK-3
uggauNuuguuugacagc	1	1	LCK-1
ugNaucuuguuugacagc	1	1	LCK-1
uggaucuuguuugacagc	2286	0	LT-1
uggCucuuguuugacagc	1	1	JCK-1
uggaucuuguuugacagU	1	1	JCK-1
uggaucuuguuuAacagc	1	1	LCK-3
uggaucuuguuugacCgc	2	1	LT-1
uggCucuuguuugacagc	53	1	LT-2
uggCucuuguuugacagc	2	1	JCK-2
uggaucuuguuugacagc	1447	0	LCK-1
uggaucuuguuuNacagc	3	1	LCK-1
uNgaucuuguuugacagc	1	1	LT-3
uggaucuuguuugacagc	1080	0	JCK-1
ugAaucuuguuugacagc	1	1	LT-3
uggaucuuguuugacGgc	1	1	LT-2
uggaucuuguuugaGagc	4	1	LCK-1
uAgaucuuguuugacagc	4	1	LT-3
uggaucuuguuugCcagc	4	1	JCK-2
uggaucuuguuugNcagc	6	1	LCK-1
uggaucuuguuugacagA	1	1	LCK-1
uggaucuuguuugCcagc	2	1	LCK-1
uggGucuuguuugacagc	1	1	LT-2
uggaucuuguuugCcagc	4	1	LT-3
uggaucuuAuuugacagc	3	1	LT-1
uggaucuuguuugacagA	1	1	JCK-3
uggaucuuguuugacagc	6351	0	LCK-3
uggaucuuguuugacCgc	6	1	LT-2
uggaucuuAuuugacagc	1	1	LT-3
uggaucuuguuugacagc	1573	0	LCK-2
uggaucuuguCugacagc	2	1	JCK-2
uggaucuuguuugCcagc	3	1	JCK-1

Mature Star

uccaucuuguucguuuacccucuggaucuuguuugacagcacucucuccuaucuuguuugacugaugaugaugggagugcugucgacaagaucuagaagguacugucgaauaagauacagauacggaggaaggaucaacuuuaccuacucucacacaaucuucuucauauucucuucaauuuuuauuuuuuuauuguucguauuuuuuuucccagauuagauuucccuguuaaucaccaugguauuaaca			
......................uggCucuuguuugacagc	2	1	JT-3
......................ugNaucuuguuugacagc	7	1	LT-2
......................ugAaucuuguuugacagc	1	1	JCK-2
......................uggaucuuguuugacagA	3	1	JCK-1
......................uggaucuuguuugacagc	2328	0	LT-3
......................uggaucuuguuugacagU	21	1	LT-2
......................uggCucuuguuugacagc	2	1	JT-2
......................uggaucuuguuugacagU	3	1	JCK-2
......................uNgaucuuguuugacagc	1	1	JCK-3
......................uggaucuuguuugGcagc	2	1	LCK-1
......................uNgaucuuguuugacagc	1	1	LCK-3
......................uAgaucuuguuugacagc	1	1	LT-1
......................uUgaucuuguuugacagc	1	1	JCK-2
......................uggaucuuguuugacaCc	2	1	JCK-3
......................uAgaucuuguuugacagc	1	1	LCK-2
......................uggaucuuguuugacagA	6	1	LT-3
......................uNgaucuuguuugacagc	3	1	JCK-2
......................uggaucuuguuugacagc	419	0	JT-3
......................uggaucuuguuuAacagc	1	1	LCK-2
......................ugAaucuuguuugacagc	3	1	LCK-3
......................Cggaucuuguuugacagc	1	1	LT-2
......................uggaucuuguuugacagU	9	1	LT-1
......................uNgaucuuguuugacagc	1	1	JT-1
......................uUgaucuuguuugacagc	1	1	LT-2
......................uggaucuuAuuugacagc	1	1	JCK-1
......................uggaucuuguuugGcagca	1	1	JT-2
......................uggaucuuguuugacagcC	5	1	JCK-2
......................uAgaucuuguuugacagca	1	1	LT-2
......................uggaucuuguuugaAagca	1	1	LCK-2
......................uggCucuuguuugacagca	5	1	JT-1
......................uggaucGuguuugacagca	1	1	JT-3
......................uggaucuuguuugacagUa	18	1	LCK-3
......................uNgaucuuguuugacagca	1	1	JCK-3
......................uggaucuuAuuugacagca	1	1	LCK-3
......................uggGucuuguuugacagca	1	1	LCK-2
......................uggaucuuguuNgacagca	1	1	JT-2
......................uggaucCuguuugacagca	1	1	LCK-1
......................uNgaucuuguuugacagca	2	1	JCK-2
......................uggaucuCguuugacagca	1	1	LT-1
......................uAgaucuuguuugacagca	1	1	JT-1
......................ugNaucuuguuugacagca	5	1	LCK-1
......................uggaucuuguuugacagca	3633	0	LCK-2
......................uggaucuuguuugacagcG	2	1	JT-2
......................uggaucuuguuugacagcC	7	1	LCK-1
......................uUgaucuuguuugacagca	5	1	JT-3
......................uggaucuuguuugacagca	3109	0	LCK-3
......................uggaucGuguuugacagca	1	1	LCK-2
......................uNgaucuuguuugacagca	2	1	JCK-1
......................uggaucuuguuugacagca	980	0	JT-2
......................uggaucuuguuugacagcU	718	1	JCK-1
......................uggaucuuguuugacagUa	24	1	JCK-2
......................uggaucuuguuugacagcU	381	1	JT-3
......................uggaucuuguuugaGagca	4	1	LCK-1
......................uggaucuuguuugacagUa	29	1	LT-2
......................uggaucuuguuugacagAa	1	1	LT-3
......................uggCucuuguuugacagca	2	1	JCK-1
......................ugAaucuuguuugacagca	1	1	LT-3
......................ugAaucuuguuugacagca	1	1	JCK-3
......................uggaucuuguuugacagcC	21	1	LT-3
......................uggaucuuguuugacagUa	11	1	JCK-3
......................uggCucuuguuugacagca	3	1	JT-2
......................uggaucuuguuugacagcC	20	1	LT-2
......................ugUaucuuguuugacagca	1	1	LCK-2
......................uggaucuuguuGgacagca	9	1	LCK-3
......................uNgaucuuguuugacagca	1	1	LCK-3
......................uggaucuuguuNgacagca	5	1	JCK-1
......................uggaucuuguuugacCgca	1	1	JT-1
......................uggaucuuguuugacagcG	11	1	LT-3
......................uAgaucuuguuugacagca	1	1	LCK-2
......................uggaucuuUuuugacagca	1	1	LCK-2

```
                      Mature                                                  Star
uccaucuuguucguuuacccucuggaucuuguuugacagcacucucuccuaucuuguuugacugaugaugaugggagugcugucgacaagaucuagaagguacugucgaauaagauacagauacggaggaaggaucaacuuuaccuacucucacacaaucuucuucauauucucuucaauuuuuauuuuuuuauuguucguauuuuuuuucccagauuagauuucccuguuaaucaccaugguauuaaca
......................uggCucuuguuugacagca..................................................................................................................................................................................................................    4      1      LCK-3
......................uggaucuuguuugacagUa..................................................................................................................................................................................................................    11     1      JT-2
......................uggaucuuguuugacagcC..................................................................................................................................................................................................................    3      1      JT-1
......................uggaucuuguuugacagcU..................................................................................................................................................................................................................    1354   1      LT-1
......................uggauNuuguuugacagca..................................................................................................................................................................................................................    10     1      LCK-1
......................uggaucuuguuugacagca..................................................................................................................................................................................................................    2333   0      LT-2
......................ugAaucuuguuugacagca..................................................................................................................................................................................................................    1      1      JT-1
......................uggaucuuguuugacagcG..................................................................................................................................................................................................................    15     1      LT-2
......................uggaucuugCuugacagca..................................................................................................................................................................................................................    1      1      JT-1
......................uggaucuuguuugacagcC..................................................................................................................................................................................................................    14     1      LT-1
......................uggaucuuguuugacagca..................................................................................................................................................................................................................    858    0      LT-1
......................uggaucuuguuugCcagca..................................................................................................................................................................................................................    2      1      LCK-3
......................uggaucuugNuugacagca..................................................................................................................................................................................................................    1      1      LCK-1
......................uggaucuuguuugCcagca..................................................................................................................................................................................................................    5      1      JCK-2
......................uggCucuuguuugacagca..................................................................................................................................................................................................................    5      1      LCK-1
......................uggaucuuguuugacagUa..................................................................................................................................................................................................................    1      1      LCK-2
......................uggaucuCguuugacagca..................................................................................................................................................................................................................    1      1      LT-2
......................uNgaucuuguuugacagca..................................................................................................................................................................................................................    3      1      JT-1
......................uggaucuuguuugacagUa..................................................................................................................................................................................................................    12     1      JCK-1
......................uggaucuuguuugacagUa..................................................................................................................................................................................................................    3      1      LT-1
......................uggaucuuguuugNcagca..................................................................................................................................................................................................................    9      1      LCK-1
......................uUgaucuuguuugacagca..................................................................................................................................................................................................................    5      1      JT-2
......................uggaucuuguuugacagcG..................................................................................................................................................................................................................    12     1      LT-1
......................uggaucuuguuugacagcU..................................................................................................................................................................................................................    735    1      JT-1
......................uggaucCuguuugacagca..................................................................................................................................................................................................................    1      1      LT-3
......................uggaucuuguuugacagcU..................................................................................................................................................................................................................    835    1      JCK-3
......................uUgaucuuguuugacagca..................................................................................................................................................................................................................    1      1      LT-3
......................uggaucuuguuugacagca..................................................................................................................................................................................................................    3565   0      LCK-1
......................uggaucuuguuugacagcU..................................................................................................................................................................................................................    279    1      JT-2
......................uggCucuuguuugacagca..................................................................................................................................................................................................................    7      1      LCK-2
......................uggaucuuguuugacagcU..................................................................................................................................................................................................................    1372   1      LCK-1
......................uggaucuuguuugacagcU..................................................................................................................................................................................................................    2784   1      LT-2
......................uggaucuuguuugacagca..................................................................................................................................................................................................................    1294   0      JT-3
......................uggauUuuguuugacagca..................................................................................................................................................................................................................    1      1      LT-2
......................uggaucuuguuugacCgca..................................................................................................................................................................................................................    1      1      LT-2
......................uggaucuCguuugacagca..................................................................................................................................................................................................................    1      1      JT-1
......................uggaCcuuguuugacagca..................................................................................................................................................................................................................    1      1      LT-2
......................uggaucuuguuugCcagca..................................................................................................................................................................................................................    11     1      LT-2
......................uggaucuuguuugacagcC..................................................................................................................................................................................................................    21     1      LCK-3
......................uggCucuuguuugacagca..................................................................................................................................................................................................................    3      1      JCK-3
......................uggaucuuguuugCcagca..................................................................................................................................................................................................................    6      1      LCK-2
......................uggNucuuguuugacagca..................................................................................................................................................................................................................    1      1      LCK-1
......................uggaucuuguuugCcagca..................................................................................................................................................................................................................    2      1      LT-3
......................uggaucuuguuugacCgca..................................................................................................................................................................................................................    3      1      LCK-3
......................uggaucuuguuugacagca..................................................................................................................................................................................................................    1105   0      JCK-3
......................uggaucuuguuugacagcC..................................................................................................................................................................................................................    2      1      JCK-1
......................uggaucuuguuugacagUa..................................................................................................................................................................................................................    11     1      LT-3
......................uAgaucuuguuugacagca..................................................................................................................................................................................................................    3      1      LCK-3
......................uggaucuuguuugGcagca..................................................................................................................................................................................................................    1      1      LT-2
......................uggaucuuguuugaAagca..................................................................................................................................................................................................................    1      1      LCK-3
......................uggaucuuguuugGcagca..................................................................................................................................................................................................................    2      1      LCK-1
......................uggaucuuAuuugacagca..................................................................................................................................................................................................................    1      1      LCK-2
......................uggaucuuguuugCcagca..................................................................................................................................................................................................................    2      1      JCK-1
......................uggaucuuguuugaNagca..................................................................................................................................................................................................................    11     1      LCK-1
......................uggaucuuguuugacCgca..................................................................................................................................................................................................................    1      1      JCK-1
......................uggaucuuguuugacCgca..................................................................................................................................................................................................................    2      1      LCK-1
......................uggaucuuguuugacagcC..................................................................................................................................................................................................................    8      1      LCK-2
......................uggaucuuguuugacagca..................................................................................................................................................................................................................    1426   0      JT-1
......................uggaucuuguuugacCgca..................................................................................................................................................................................................................    2      1      JCK-2
......................uggaucuuguuugacagcU..................................................................................................................................................................................................................    1503   1      LCK-2
......................uggaucuuguuugacCgca..................................................................................................................................................................................................................    2      1      JT-3
......................uggCucuuguuugacagca..................................................................................................................................................................................................................    2      1      LT-1
......................uggCucuuguuugacagca..................................................................................................................................................................................................................    4      1      JCK-2
......................uggaucuuguuugacCgca..................................................................................................................................................................................................................    2      1      LT-3
......................uggaucuuguuugacagca..................................................................................................................................................................................................................    1154   0      JCK-1
......................uggaucuuguuugacagcG..................................................................................................................................................................................................................    11     1      LCK-3
......................uggCucuuguuugacagca..................................................................................................................................................................................................................    12     1      LT-2
......................uggauAuuguuugacagca..................................................................................................................................................................................................................    1      1      JCK-3
......................uggaucuCguuugacagca..................................................................................................................................................................................................................    1      1      LCK-2
......................uggaucuuguuugacagcU..................................................................................................................................................................................................................    1214   1      JCK-2
```

Mature Star

uccaucuuguucguuuacccucuggaucuuguuugacagcacucucuccuaucuuguuugacugaugaugaugggagugcugucgacaagaucuagaagguacugucgaauaagauacagauacggaggaaggaucaacuuuaccuacucucacacaaucuucuucauauucucuucaauuuuuauuuuuuuauuguucguauuuuuuuucccagauuagauuucccuguuaaucaccaugguauuaaca

......................uggaucuuguuugacagcG	2	1	JCK-2
......................uggaucuuguuugacagUa	10	1	JT-3
......................uggaucuuguuugacagcG	6	1	JCK-1
......................uggaucuuguuNgacagca	2	1	JT-1
......................uggGucuuguuugacagca	1	1	JCK-1
......................uAgaucuuguuugacagca	1	1	LT-3
......................uggaucuuguuugacagca	1862	0	JCK-2
......................uggaucuuguuuUacagca	1	1	LCK-3
......................uggaucuuguuugacagcG	2	1	JCK-3
......................ugNaucuuguuugacagca	9	1	LT-2
......................uggCucuuguuugacagca	2	1	LT-3
......................uggaucuuguuugacagca	1490	0	LT-3
......................uggaucuuguuugacagcC	6	1	JCK-3
......................uggaucCuguuugacagca	1	1	LT-2
......................uggaucuuguuugacagcU	4465	1	LCK-3
......................uggCucuuguuugacagca	2	1	JT-3
......................Cggaucuuguuugacagca	1	1	LCK-3
......................uggaucuuguuuNacagca	2	1	LCK-1
......................uggaucuuguuugCcagca	3	1	LCK-1
......................uggaucuuguuugacCgca	1	1	JCK-3
......................uggaucuuguuugCcagca	1	1	LT-1
......................uggaucuuguuugacagcU	2081	1	LT-3
......................uggaucuuguuugacagcac	216	0	JT-1
......................uggaucuuguuugCcagcac	1	1	LCK-3
......................uggaucuuguuugacagcac	545	0	LCK-1
......................uggaucuuguuugCcagcac	1	1	LT-1
......................uggaucuuguuugacagcUc	26	1	LCK-1
......................uggaucuuguuugacagcaU	16	1	JT-1
......................uggaucuuguuugacagcaA	1	1	JT-3
......................uggaucuuguuugacagcaU	22	1	LT-1
......................uggaCcuuguuugacagcac	1	1	JCK-2
......................uNgaucuuguuugacagcac	1	1	LCK-3
......................uggCucuuguuugacagcac	1	1	LCK-2
......................uggaucuuguuugacagcaU	26	1	LT-2
......................uggaucuuguuugacagcCc	1	1	JT-1
......................uNgaucuuguuugacagcac	1	1	JCK-1
......................uggCucuuguuugacagcac	5	1	LCK-3
......................uggaucuuguuugacagcCc	1	1	LT-2
......................uggaucuuguuugacagcac	982	0	LT-2
......................uAgaucuuguuugacagcac	1	1	LCK-1
......................uggaucuuguuugacagcNc	1	1	JCK-1
......................uggaucuuguuugaNagcac	2	1	LCK-1
......................uggaucuuguuugacagcaU	46	1	JCK-2
......................uggaucuuguuugacagcaA	4	1	JCK-3
......................uggCucuuguuugacagcac	2	1	LT-3
......................uggaucuuguuugacagcCc	2	1	LT-1
......................uggaucuuguuugacagcaU	8	1	JCK-1
......................uggaucuuguuugacagcaU	26	1	JCK-3
......................Aggaucuuguuugacagcac	1	1	LCK-3
......................uggaucuuguuugCcagcac	2	1	JT-1
......................uggaucuuguuugacagcUc	51	1	LT-3
......................uggaucuuguuugCcagcac	1	1	LT-3
......................uggaucuuguuugNcagcac	2	1	LCK-1
......................uggaucuuguuugGcagcac	1	1	LCK-1
......................uggauNuuguuugacagcac	1	1	LCK-1
......................uggaucuuguuugacGgcac	1	1	LT-2
......................uggaucuuguuugacagUac	1	1	JCK-3
......................uggaucuuguuugacagcaA	3	1	LT-3
......................uAgaucuuguuugacagcac	1	1	LCK-3
......................uggaucuugCuugacagcac	1	1	LT-2
......................uggaucuuguuugacagcUc	6	1	JCK-1
......................uggCucuuguuugacagcac	1	1	JCK-1
......................uggaucuuguuNgacagcac	1	1	JT-2
......................uggaucuuguuugacagcac	666	0	LT-1
......................uggaucuuguuugacagcac	1318	0	LCK-3
......................uggaucuuguuugacCgcac	2	1	JCK-2
......................uggaucuuguuugacagcUc	7	1	JCK-3
......................uggaucuuguuugacCgcac	3	1	LT-3
......................uggaucuuguuugacagcUc	63	1	LCK-3
......................uggaucuuguuugacagcaN	2	1	JCK-1

```
                      Mature                                                  Star
uccaucuuguucguuuacccucuggaucuuguuugacagcacucucuccuaucuuguuugacugaugaugaugggagugcugucgacaagaucuagaagguacugucgaauaagauacagauacggaggaaggaucaacuuuaccuacucucacacaaucuucuucauauucucuucaauuuuuauuuuuuuauuguucguauuuuuuuucccagauuagauuucccuguuaaucaccaugguauuaaca
......................ugNaucuuguuugacagcac................................................................................................................................................................................................ 3 1 LT-2
......................uggaucuuguuugacagcac................................................................................................................................................................................................ 899 0 JCK-2
......................uggaucuuguuugacagcac................................................................................................................................................................................................ 249 0 JT-3
......................uggaucuuguuugCcagcac................................................................................................................................................................................................ 3 1 JCK-2
......................uggaucuuguuugacagcaA................................................................................................................................................................................................ 1 1 LCK-2
......................uggaucuuguuugacagcac................................................................................................................................................................................................ 525 0 LCK-2
......................uAgaucuuguuugacagcac................................................................................................................................................................................................ 4 1 LT-2
......................uggaucuuguuuAacagcac................................................................................................................................................................................................ 1 1 LCK-2
......................uggaucuuguuugacagcac................................................................................................................................................................................................ 715 0 LT-3
......................uggGucuuguuugacagcac................................................................................................................................................................................................ 2 1 LT-2
......................uggaucuuguuugacagcaU................................................................................................................................................................................................ 66 1 LCK-3
......................uggaucuuguuugacagcUc................................................................................................................................................................................................ 15 1 JCK-2
......................uggaCcuuguuugacagcac................................................................................................................................................................................................ 1 1 JCK-3
......................uggaucuuguuugacagcac................................................................................................................................................................................................ 170 0 JT-2
......................uggaucuuguuugacagcaA................................................................................................................................................................................................ 1 1 JCK-2
......................uggaucuuguuugacagcac................................................................................................................................................................................................ 687 0 JCK-3
......................uAgaucuuguuugacagcac................................................................................................................................................................................................ 1 1 LT-1
......................uggaucuuUuuugacagcac................................................................................................................................................................................................ 1 1 LT-3
......................uggaucuuguuugGcagcac................................................................................................................................................................................................ 1 1 LT-3
......................uggaCcuuguuugacagcac................................................................................................................................................................................................ 1 1 JT-1
......................uggCucuuguuugacagcac................................................................................................................................................................................................ 1 1 LT-1
......................uggaucuuguuugacagcaA................................................................................................................................................................................................ 3 1 JCK-1
......................uggaucuuguuugacagcac................................................................................................................................................................................................ 586 0 JCK-1
......................uggCucuuguuugacagcac................................................................................................................................................................................................ 2 1 JCK-2
......................uggaucuuguuugacagcaG................................................................................................................................................................................................ 1 1 LT-1
......................uggaucuuguCugacagcac................................................................................................................................................................................................ 1 1 JCK-2
......................uggaucuuguuugacagcUc................................................................................................................................................................................................ 77 1 LT-2
......................uggaucuuguuugacagcUc................................................................................................................................................................................................ 31 1 LCK-2
......................uggaucuuguuugacCgcac................................................................................................................................................................................................ 1 1 LT-2
......................uggaucuuguuugCcagcac................................................................................................................................................................................................ 4 1 LT-2
......................ugAaucuuguuugacagcac................................................................................................................................................................................................ 1 1 JCK-1
......................uggCucuuguuugacagcac................................................................................................................................................................................................ 1 1 LCK-1
......................uggCucuuguuugacagcac................................................................................................................................................................................................ 2 1 JT-3
......................uggCucuuguuugacagcac................................................................................................................................................................................................ 9 1 LT-2
......................uggaucuuguuugacagcUc................................................................................................................................................................................................ 36 1 LT-1
......................uggaucuuguuugacagcaU................................................................................................................................................................................................ 26 1 LT-3
......................uggaucuuguuugCcagcac................................................................................................................................................................................................ 1 1 JT-2
......................uggaucuuCuuugacagcac................................................................................................................................................................................................ 1 1 LCK-3
......................uggaucuuguuugCcagcac................................................................................................................................................................................................ 1 1 JCK-3
......................uggaucuuguuugacagcGc................................................................................................................................................................................................ 1 1 LCK-3
......................uggaucuuguuugCcagcac................................................................................................................................................................................................ 1 1 JCK-1
......................uggaucuugCuugacagcacu............................................................................................................................................................................................... 4 1 LCK-3
......................uggauUuuguuugacagcacu............................................................................................................................................................................................... 4 1 JCK-3
......................uggaucuuguuugacagcGcu............................................................................................................................................................................................... 2 1 JCK-2
......................uggaucuuguuuAacagcacu............................................................................................................................................................................................... 3 1 LT-1
......................uggaucuuguuGgacagcacu............................................................................................................................................................................................... 1 1 JCK-2
......................uggaucuuCuuugacagcacu............................................................................................................................................................................................... 1 1 JCK-3
......................uggauUuuguuugacagcacu............................................................................................................................................................................................... 2 1 LT-3
......................uggaCcuuguuugacagcacu............................................................................................................................................................................................... 1 1 LCK-3
......................uggaucuuguuugacCgcacu............................................................................................................................................................................................... 19 1 JCK-1
......................uggaucuugCuugacagcacu............................................................................................................................................................................................... 2 1 LT-1
......................uggaucuuguuugacaCcacu............................................................................................................................................................................................... 1 1 JT-1
......................uNgaucuuguuugacagcacu............................................................................................................................................................................................... 104 1 JCK-2
......................uggauGuuguuugacagcacu............................................................................................................................................................................................... 1 1 JCK-3
......................uggaucuuguCugacagcacu............................................................................................................................................................................................... 7 1 JCK-3
......................uggaucuuguuugacagcacG............................................................................................................................................................................................... 12 1 LT-3
......................uggaucuuguuugacagUacu............................................................................................................................................................................................... 18 1 JCK-1
......................uggaucuuguuugacagcaUu............................................................................................................................................................................................... 102 1 LCK-3
......................uggaucuuguuugacagcaNu............................................................................................................................................................................................... 42 1 JCK-1
......................uggaucuuguuugacagcacu............................................................................................................................................................................................... 1709 0 JT-1
......................uAgaucuuguuugacagcacu............................................................................................................................................................................................... 15 1 JCK-3
......................uggaucuuguuGgacagcacu............................................................................................................................................................................................... 1 1 LT-3
......................uggaucuuguCugacagcacu............................................................................................................................................................................................... 1 1 LT-3
......................uggaucuuguuugacagcaGu............................................................................................................................................................................................... 2 1 JCK-1
......................uggaucuuguuugacagcacu............................................................................................................................................................................................... 5029 0 LCK-2
......................uggCucuuguuugacagcacu............................................................................................................................................................................................... 65 1 LT-1
......................uggaCcuuguuugacagcacu............................................................................................................................................................................................... 14 1 JCK-2
......................uggaucuuguuugacagcaAu............................................................................................................................................................................................... 4 1 JT-3
......................uggaucuuguuugCcagcacu............................................................................................................................................................................................... 86 1 JCK-1
......................uggaucuugCuugacagcacu............................................................................................................................................................................................... 6 1 JCK-3
```

```
                      Mature                                                    Star
uccaucuuguucguuuacccucuggaucuuguuugacagcacucucuccuaucuuguuugacugaugaugaugggagugcugucgacaagaucuagaagguacugucgaauaagauacagauacggaggaaggaucaacuuuaccuacucucacacaaucuucuucauauucucuucaauuuuuauuuuuuuauuguucguauuuuuuuucccagauuagauuucccuguuaaucaccaugguauuaaca
......................uggauNuuguuugacagcacu...............................................................................................................................................................................    3      1    LCK-1
......................uggaucuuguuugacagcacu...............................................................................................................................................................................    20839  0    LT-2
......................uggaucuuguuugacagcaUu...............................................................................................................................................................................    21     1    JT-3
......................uggaucuuguuugacagcGcu...............................................................................................................................................................................    3      1    LT-2
......................uggaucuuguuugacagGacu...............................................................................................................................................................................    2      1    JCK-3
......................uNgaucuuguuugacagcacu...............................................................................................................................................................................    1      1    LCK-1
......................uggNucuuguuugacagcacu...............................................................................................................................................................................    22     1    JCK-1
......................uggauNuuguuugacagcacu...............................................................................................................................................................................    1      1    JCK-2
......................uggaucuuguuugacagAacu...............................................................................................................................................................................    3      1    LT-1
......................uggaucuuguuugGcagcacu...............................................................................................................................................................................    3      1    JCK-1
......................uggaucuuguuugacagcacu...............................................................................................................................................................................    38814  0    JCK-1
......................uUgaucuuguuugacagcacu...............................................................................................................................................................................    1      1    JT-3
......................Cggaucuuguuugacagcacu...............................................................................................................................................................................    9      1    LT-3
......................uggaucuuguuuCacagcacu...............................................................................................................................................................................    1      1    JCK-2
......................uggaucuuguuugacagcaUu...............................................................................................................................................................................    22     1    JT-2
......................uggaucCuguuugacagcacu...............................................................................................................................................................................    12     1    JCK-2
......................uggaucuuguuugacagUacu...............................................................................................................................................................................    5      1    LT-3
......................uggaucuuguuuAacagcacu...............................................................................................................................................................................    2      1    LT-2
......................uNgaucuuguuugacagcacu...............................................................................................................................................................................    4      1    JT-1
......................uggaucuuguuugacagcCcu...............................................................................................................................................................................    31     1    LT-3
......................uggaucuuguuugGcagcacu...............................................................................................................................................................................    2      1    LT-1
......................uUgaucuuguuugacagcacu...............................................................................................................................................................................    4      1    JCK-2
......................uggaucuuguCugacagcacu...............................................................................................................................................................................    3      1    LT-1
......................uggaucuuguuugacGgcacu...............................................................................................................................................................................    1      1    JT-3
......................uggaucuuguuNgacagcacu...............................................................................................................................................................................    1      1    JCK-2
......................uAgaucuuguuugacagcacu...............................................................................................................................................................................    8      1    JCK-2
......................uUgaucuuguuugacagcacu...............................................................................................................................................................................    2      1    LCK-3
......................uggaucuuguuugGcagcacu...............................................................................................................................................................................    3      1    JCK-3
......................uggaCcuuguuugacagcacu...............................................................................................................................................................................    3      1    LT-1
......................Cggaucuuguuugacagcacu...............................................................................................................................................................................    11     1    JCK-2
......................uggaucuuguuugacagGacu...............................................................................................................................................................................    24     1    JCK-1
......................uggaucuuAuuugacagcacu...............................................................................................................................................................................    3      1    LT-3
......................ugAaucuuguuugacagcacu...............................................................................................................................................................................    3      1    JCK-1
......................uggaucuugGuugacagcacu...............................................................................................................................................................................    1      1    JCK-1
......................uggaucuuguuugacGgcacu...............................................................................................................................................................................    2      1    LT-1
......................uggaucuCguuugacagcacu...............................................................................................................................................................................    10     1    JCK-2
......................uggaucuuguuugacagcaUu...............................................................................................................................................................................    68     1    LT-2
......................ugAaucuuguuugacagcacu...............................................................................................................................................................................    11     1    LT-3
......................uggaucuuguuugacCgcacu...............................................................................................................................................................................    26     1    LT-2
......................uggaCcuuguuugacagcacu...............................................................................................................................................................................    4      1    LT-3
......................Gggaucuuguuugacagcacu...............................................................................................................................................................................    1      1    JCK-2
......................uggaucuuguuGgacagcacu...............................................................................................................................................................................    1      1    JCK-3
......................uggauUuuguuugacagcacu...............................................................................................................................................................................    3      1    JCK-1
......................uggaucuuguuugacagcacu...............................................................................................................................................................................    658    0    JT-2
......................uggaucuuguuugacagUacu...............................................................................................................................................................................    11     1    LT-2
......................uggaucuuguGugacagcacu...............................................................................................................................................................................    2      1    LT-2
......................uggaucuuguuugacagcUcu...............................................................................................................................................................................    6      1    LT-2
......................uggaucuuguuugacagcaAu...............................................................................................................................................................................    10     1    JCK-2
......................uggCucuuguuugacagcacu...............................................................................................................................................................................    92     1    JCK-1
......................uggaucuuUuuugacagcacu...............................................................................................................................................................................    1      1    LT-3
......................uggaucuuguuugacagcUcu...............................................................................................................................................................................    1      1    JCK-2
......................uggGucuuguuugacagcacu...............................................................................................................................................................................    5      1    JCK-3
......................uggaucuuguuugaAagcacu...............................................................................................................................................................................    2      1    JCK-1
......................uggaucuuguCugacagcacu...............................................................................................................................................................................    5      1    JT-2
......................uggaucuCguuugacagcacu...............................................................................................................................................................................    3      1    LCK-3
......................uAgaucuuguuugacagcacu...............................................................................................................................................................................    5      1    JCK-1
......................uggaucuuCuuugacagcacu...............................................................................................................................................................................    1      1    JCK-2
......................uggaucCuguuugacagcacu...............................................................................................................................................................................    10     1    JCK-1
......................uggaucuuguuugacagcCcu...............................................................................................................................................................................    4      1    LCK-2
......................ugUaucuuguuugacagcacu...............................................................................................................................................................................    3      1    LT-3
......................uggaucuuguuGgacagcacu...............................................................................................................................................................................    10     1    JCK-1
......................uggaucuuguuugGcagcacu...............................................................................................................................................................................    1      1    LT-2
......................uggaucuuguuugaNagcacu...............................................................................................................................................................................    17     1    LCK-1
......................uggaucuuguuugacagcaAu...............................................................................................................................................................................    3      1    JCK-1
......................uAgaucuuguuugacagcacu...............................................................................................................................................................................    22     1    LT-3
......................uggaucuuguuugaGagcacu...............................................................................................................................................................................    8      1    LCK-1
......................uggaucCuguuugacagcacu...............................................................................................................................................................................    4      1    LT-1
......................uggaucuuNuuugacagcacu...............................................................................................................................................................................    1      1    JCK-3
......................uggaucGuguuugacagcacu...............................................................................................................................................................................    1      1    LT-1
......................uggaCcuuguuugacagcacu...............................................................................................................................................................................    4      1    JCK-1
```

Mature Star

uccaucuuguucguuuacccucuggaucuuguuugacagcacucucuccuaucuuguuugacugaugaugaugggagugcugucgacaagaucuagaagguacugucgaauaagauacagauacggaggaaggaucaacuuuaccuacucucacacaaucuucuucauauucucuucaauuuuuauuuuuuuauuguucguauuuuuuuucccagauuagauuucccuguuaaucaccaugguauuaaca			
......................uggaucuuguuuUacagcacu...	2	1	JCK-3
......................uggaucuuguuugCcagcacu...	1	1	JT-3
......................uggaucuuguuugCcagcacu...	61	1	LT-1
......................uggaucuuguuugCcagcacu...	7	1	LCK-2
......................uggaucuuguuuAacagcacu...	9	1	JCK-2
......................uggaucuuguuugacagUacu...	1	1	JT-2
......................ugAaucuuguuugacagcacu...	3	1	LCK-2
......................uggCucuuguuugacagcacu...	118	1	JCK-3
......................uggaGcuuguuugacagcacu...	2	1	JCK-3
......................uggaucuugCuugacagcacu...	4	1	LT-2
......................uggaucuuguuAgacagcacu...	1	1	JCK-2
......................uggaucuuguuugacagcCcu...	16	1	LCK-3
......................uggaucCuguuugacagcacu...	2	1	LT-2
......................ugUaucuuguuugacagcacu...	2	1	JCK-1
......................uggaucuuguuugacagcCcu...	34	1	LT-2
......................Cggaucuuguuugacagcacu...	4	1	LT-2
......................uggaucuuAuuugacagcacu...	6	1	LT-1
......................uggaucuuguuAgacagcacu...	1	1	JCK-1
......................uggaucuuguuugacagcacu...	27651	0	LT-3
......................uggaucuuguuugacGgcacu...	3	1	LT-3
......................uggaucuuguuugCcagcacu...	5	1	JT-1
......................uggaucuuguuugacagcaUu...	1732	1	JCK-3
......................uggaucuuguuugacagcGcu...	1	1	JT-3
......................uggaucuuguuugacagcacG...	3	1	LT-2
......................uggaucuuguuugacagGacu...	2	1	JCK-2
......................uggaucuuguuNgacagcacu...	121	1	JCK-1
......................uggaucuuguuugacaCcacu...	5	1	JCK-3
......................uCgaucuuguuugacagcacu...	1	1	JCK-3
......................uggaucuuguuugacCgcacu...	5	1	LCK-2
......................Cggaucuuguuugacagcacu...	6	1	JCK-1
......................uggauUuuguuugacagcacu...	1	1	LT-2
......................uggaucuuguuugacaCcacu...	5	1	JCK-1
......................uNgaucuuguuugacagcacu...	2	1	LT-3
......................uggCucuuguuugacagcacu...	2	1	JT-2
......................uggaucuuguuuUacagcacu...	4	1	LT-1
......................uggaucuuguuugacagcaUu...	2361	1	JCK-2
......................ugNaucuuguuugacagcacu...	1	1	LCK-1
......................uggaucCuguuugacagcacu...	2	1	LT-3
......................uggauAuuguuugacagcacu...	1	1	JCK-3
......................uggaucuuguuugacagcaAu...	5	1	JT-2
......................uggaucuuguuuUacagcacu...	3	1	LT-3
......................uggauUuuguuugacagcacu...	11	1	JCK-2
......................uggaucuuAuuugacagcacu...	6	1	JCK-1
......................uggaucuuguuuAacagcacu...	1	1	JT-1
......................uNgaucuuguuugacagcacu...	3	1	LT-1
......................uggaucuuguuugacCgcacu...	28	1	JCK-3
......................uggaucuuUuuugacagcacu...	3	1	JCK-1
......................uggaucuuguuuAacagcacu...	1	1	LT-3
......................uggaucuuguuugacagAacu...	1	1	JCK-2
......................uggaucuuguuugUcagcacu...	2	1	JCK-2
......................uggaucuuguuugacagcacN...	22	1	JCK-1
......................uggaucuuguuugCcagcacu...	5	1	LCK-1
......................uggaucuuAuuugacagcacu...	9	1	LT-2
......................uggaucuuguuugacagcacG...	13	1	JCK-3
......................uAgaucuuguuugacagcacu...	4	1	LCK-2
......................ugCaucuuguuugacagcacu...	2	1	LT-2
......................uggaucuuguuugacagcUcu...	5	1	LT-1
......................uggaucuuguuuUacagcacu...	2	1	LT-2
......................uggaucuuguuugCcagcacu...	159	1	JCK-2
......................uggCucuuguuugacagcacu...	152	1	JCK-2
......................uggaucuuguuugacagcacG...	10	1	LT-1
......................uggaucuuguuugacagNacu...	3	1	JCK-3
......................uggaucuuguuuAacagcacu...	7	1	JCK-3
......................uggaucuuguuugacagcacG...	10	1	JCK-1
......................Cggaucuuguuugacagcacu...	7	1	JCK-3
......................uggCucuuguuugacagcacu...	200	1	LT-2
......................uCgaucuuguuugacagcacu...	1	1	LCK-3
......................ugAaucuuguuugacagcacu...	10	1	LT-2
......................uggaucuugCuugacagcacu...	1	1	LT-3
......................uggaucuuUuuugacagcacu...	1	1	LCK-3

```
                      Mature                                                      Star
uccaucuuguucguuuacccucuggaucuuguuugacagcacucucuccuaucuuguuugacugaugaugaugggagugcugucgacaagaucuagaagguacugucgaauaagauacagauacggaggaaggaucaacuuuaccuacucucacacaaucuucuucauauucucuucaauuuuuauuuuuuuauuguucguauuuuuuuuucccagauuagauuucccuguuaaucaccaugguauuaaca
......................uggaucuuguuugacagcCcu................................................................................................................................................................................................   74     1    JCK-2
......................uggaucuuguuuCacagcacu................................................................................................................................................................................................   2      1    JCK-1
......................uggaucuuguuugacagcaUu................................................................................................................................................................................................   55     1    JT-1
......................uggaucuuguuugacagcacu................................................................................................................................................................................................   4848   0    LCK-1
......................uggaucuuguuugacaCcacu................................................................................................................................................................................................   1      1    LT-3
......................uggaCcuuguuugacagcacu................................................................................................................................................................................................   1      1    LCK-2
......................uggaucuuguuugacagcNcu................................................................................................................................................................................................   1      1    LCK-1
......................uggCucuuguuugacagcacu................................................................................................................................................................................................   1      1    JT-3
......................uggaucuugCuugacagcacu................................................................................................................................................................................................   2      1    JCK-1
......................ugAaucuuguuugacagcacu................................................................................................................................................................................................   2      1    LCK-1
......................uggaucuuguuugaAagcacu................................................................................................................................................................................................   3      1    LT-1
......................uggaucuuguuugCcagcacu................................................................................................................................................................................................   2      1    JT-2
......................uNgaucuuguuugacagcacu................................................................................................................................................................................................   55     1    JCK-3
......................uggaucuuguGugacagcacu................................................................................................................................................................................................   15     1    JCK-1
......................uggaucuuUuuugacagcacu................................................................................................................................................................................................   6      1    JCK-3
......................uggaCcuuguuugacagcacu................................................................................................................................................................................................   13     1    JCK-3
......................uggaucuuguuugaAagcacu................................................................................................................................................................................................   5      1    JCK-3
......................uggaucuuguuugacagAacu................................................................................................................................................................................................   1      1    JCK-1
......................uggaucuugGuugacagcacu................................................................................................................................................................................................   1      1    JCK-3
......................uggauUuuguuugacagcacu................................................................................................................................................................................................   1      1    LCK-3
......................uggaucuuguuugacGgcacu................................................................................................................................................................................................   7      1    LT-2
......................uUgaucuuguuugacagcacu................................................................................................................................................................................................   7      1    LT-2
......................uggaucuugGuugacagcacu................................................................................................................................................................................................   1      1    JCK-2
......................ugUaucuuguuugacagcacu................................................................................................................................................................................................   1      1    JCK-2
......................uggaucuuguuugacagcacG................................................................................................................................................................................................   1      1    JT-2
......................uggaucuuguuugacagcacG................................................................................................................................................................................................   7      1    LCK-3
......................uggaucuuguuugacGgcacu................................................................................................................................................................................................   9      1    JCK-3
......................uggaucuuguuuNacagcacu................................................................................................................................................................................................   1      1    LCK-1
......................uggaucuuguuNgacagcacu................................................................................................................................................................................................   1      1    JT-1
......................uggaucuuguuugacGgcacu................................................................................................................................................................................................   7      1    JCK-1
......................uggaucuuguuugGcagcacu................................................................................................................................................................................................   5      1    LCK-1
......................uggaucuuguuugacagcaAu................................................................................................................................................................................................   1      1    LT-1
......................uggaucuuguuuUacagcacu................................................................................................................................................................................................   6      1    JCK-1
......................uggaucuuguuugacagcCcu................................................................................................................................................................................................   29     1    LT-1
......................Cggaucuuguuugacagcacu................................................................................................................................................................................................   1      1    JT-3
......................uggaucuuguuugCcagcacu................................................................................................................................................................................................   80     1    LT-2
......................uggaucuuguuugacagUacu................................................................................................................................................................................................   34     1    JCK-2
......................ugUaucuuguuugacagcacu................................................................................................................................................................................................   4      1    JCK-3
......................uggNucuuguuugacagcacu................................................................................................................................................................................................   1      1    JCK-2
......................uggaucuuguuugNcagcacu................................................................................................................................................................................................   15     1    LCK-1
......................uggaucuuguuugacGgcacu................................................................................................................................................................................................   10     1    JCK-2
......................uggaucuuguuuAacagcacu................................................................................................................................................................................................   1      1    LCK-3
......................uggaucuuguuugacagcaAu................................................................................................................................................................................................   4      1    LT-2
......................uggaucuuguuugacagcaAu................................................................................................................................................................................................   1      1    LCK-3
......................uggaucuuguuugacagcUcu................................................................................................................................................................................................   1      1    LT-3
......................ugUaucuuguuugacagcacu................................................................................................................................................................................................   2      1    LT-2
......................uAgaucuuguuugacagcacu................................................................................................................................................................................................   13     1    LCK-3
......................uAgaucuuguuugacagcacu................................................................................................................................................................................................   30     1    LT-2
......................uggCucuuguuugacagcacu................................................................................................................................................................................................   9      1    LCK-2
......................uggaCcuuguuugacagcacu................................................................................................................................................................................................   2      1    LT-2
......................uggNucuuguuugacagcacu................................................................................................................................................................................................   3      1    JCK-3
......................ugAaucuuguuugacagcacu................................................................................................................................................................................................   19     1    JCK-2
......................uggaucCuguuugacagcacu................................................................................................................................................................................................   1      1    LCK-1
......................uggaucuuguuugacCgcacu................................................................................................................................................................................................   7      1    LCK-3
......................uggaucuCguuugacagcacu................................................................................................................................................................................................   5      1    JCK-1
......................ugAaucuuguuugacagcacu................................................................................................................................................................................................   8      1    JCK-3
......................uggaucuuguuugacagcaAu................................................................................................................................................................................................   1      1    LCK-2
......................uggaucuuguuugGcagcacu................................................................................................................................................................................................   1      1    LT-3
......................uggaucuuguuugacCgcacu................................................................................................................................................................................................   3      1    JT-1
......................ugAaucuuguuugacagcacu................................................................................................................................................................................................   6      1    LT-1
......................uggaucuuguuugacaCcacu................................................................................................................................................................................................   3      1    JCK-2
......................uggaucuGguuugacagcacu................................................................................................................................................................................................   1      1    JCK-2
......................uggaucuuguuugacCgcacu................................................................................................................................................................................................   12     1    LT-1
......................uggaucuuguuugacagcNcu................................................................................................................................................................................................   22     1    JCK-1
......................uggaucuuguuugacagcacu................................................................................................................................................................................................   66730  0    JCK-2
......................uggaucuuguuugacagcCcu................................................................................................................................................................................................   53     1    JCK-1
......................uggaucuCguuugacagcacu................................................................................................................................................................................................   1      1    LCK-1
......................uggaucuuguuugCcagcacu................................................................................................................................................................................................   105    1    JCK-3
......................uggaucuuguCugacagcacu................................................................................................................................................................................................   1      1    LT-2
......................ugAaucuuguuugacagcacu................................................................................................................................................................................................   4      1    LCK-3
```

```
                      Mature                                                      Star
uccaucuuguucguuuacccucuggaucuuguuugacagcacucucuccuaucuuguuugacugaugaugaugggagugcugucgacaagaucuagaagguacugucgaauaagauacagauacggaggaaggaucaacuuuaccuacucucacacaaucuucuucauauucucuucaauuuuuauuuuuuuauuguucguauuuuuuuucccagauuagauuucccuguuaaucaccaugguauuaaca
......................uggaucuuguCugacagcacu.......................................................................................................................................................................................   6       1   JCK-1
......................uggaucuuguuuCacagcacu.......................................................................................................................................................................................   1       1   JCK-3
......................uggaucuuguuugacagcacu.......................................................................................................................................................................................   23834   0   LT-1
......................uggaucCuguuugacagcacu.......................................................................................................................................................................................   8       1   JCK-3
......................uggaucuuguuuUacagcacu.......................................................................................................................................................................................   6       1   JCK-2
......................uggaucuuguuugacagcaUu.......................................................................................................................................................................................   64      1   LT-3
......................uggaucuuguuugacagcaAu.......................................................................................................................................................................................   3       1   LT-3
......................Cggaucuuguuugacagcacu.......................................................................................................................................................................................   3       1   LT-1
......................uggaucuuguuugGcagcacu.......................................................................................................................................................................................   3       1   JCK-2
......................uggaucuuAuuugacagcacu.......................................................................................................................................................................................   1       1   LCK-1
......................uAgaucuuguuugacagcacu.......................................................................................................................................................................................   14      1   LT-1
......................ugUaucuuguuugacagcacu.......................................................................................................................................................................................   1       1   JT-1
......................ugNaucuuguuugacagcacu.......................................................................................................................................................................................   65      1   LT-2
......................uUgaucuuguuugacagcacu.......................................................................................................................................................................................   10      1   JCK-3
......................uggaucuuguuugacagcCcu.......................................................................................................................................................................................   17      1   JCK-3
......................uggaucuuUuuugacagcacu.......................................................................................................................................................................................   2       1   LT-1
......................uggaucuuguCugacagcacu.......................................................................................................................................................................................   1       1   LCK-2
......................uggGucuuguuugacagcacu.......................................................................................................................................................................................   8       1   JCK-1
......................uggaucuuguuugCcagcacu.......................................................................................................................................................................................   36      1   LCK-3
......................uggaucuuguCugacagcacu.......................................................................................................................................................................................   6       1   JCK-2
......................uggCucuuguuugacagcacu.......................................................................................................................................................................................   7       1   JT-1
......................ugUaucuuguuugacagcacu.......................................................................................................................................................................................   1       1   LCK-2
......................ugCaucuuguuugacagcacu.......................................................................................................................................................................................   1       1   LCK-3
......................uggaucuuguuugaGagcacu.......................................................................................................................................................................................   1       1   JT-1
......................uggaucuuguuAgacagcacu.......................................................................................................................................................................................   1       1   LCK-3
......................uggaucuuguuugacCgcacu.......................................................................................................................................................................................   3       1   LCK-1
......................uggaucuuAuuugacagcacu.......................................................................................................................................................................................   1       1   LCK-2
......................uggaucuuguuugacCgcacu.......................................................................................................................................................................................   11      1   LT-3
......................uggaucuuguuuAacagcacu.......................................................................................................................................................................................   1       1   LCK-1
......................uggaucuuAuuugacagcacu.......................................................................................................................................................................................   20      1   JCK-2
......................uNgaucuuguuugacagcacu.......................................................................................................................................................................................   66      1   JCK-1
......................uggaucuuguuuAacagcacu.......................................................................................................................................................................................   6       1   JCK-1
......................uggaucuuguuugacagcacG.......................................................................................................................................................................................   2       1   LCK-1
......................uggaucuuguuugacagcCcu.......................................................................................................................................................................................   4       1   LCK-1
......................uggauAuuguuugacagcacu.......................................................................................................................................................................................   2       1   JCK-2
......................uggaucuuguuugacagcaUu.......................................................................................................................................................................................   36      1   LCK-1
......................uggaucuuCuuugacagcacu.......................................................................................................................................................................................   1       1   JCK-1
......................uggaucuuguuugacagcaUu.......................................................................................................................................................................................   20      1   LT-1
......................uggaucuuguuugacagcacu.......................................................................................................................................................................................   20263   0   LCK-3
......................uggaucuuAuuugacagcacu.......................................................................................................................................................................................   4       1   JCK-3
......................uggCucuuguuugacagcacu.......................................................................................................................................................................................   8       1   LCK-1
......................uggaucGuguuugacagcacu.......................................................................................................................................................................................   2       1   JCK-1
......................uggaucuuguuugaAagcacu.......................................................................................................................................................................................   4       1   JCK-2
......................uggaucuCguuugacagcacu.......................................................................................................................................................................................   4       1   JCK-3
......................uggaucuuguuugacagUacu.......................................................................................................................................................................................   13      1   JCK-3
......................uggaucuuAuuugacagcacu.......................................................................................................................................................................................   4       1   LCK-3
......................uggaucuCguuugacagcacu.......................................................................................................................................................................................   1       1   LT-1
......................uNgaucuuguuugacagcacu.......................................................................................................................................................................................   1       1   LCK-2
......................uggaucuCguuugacagcacu.......................................................................................................................................................................................   3       1   LT-3
......................uggaucuuguuugCcagcacu.......................................................................................................................................................................................   67      1   LT-3
......................uggaucuuguuugacGgcacu.......................................................................................................................................................................................   2       1   LCK-3
......................uggaucuuguuugacCgcacu.......................................................................................................................................................................................   29      1   JCK-2
......................uggaucuuguuugacagcaAu.......................................................................................................................................................................................   3       1   JCK-3
......................uggaucuuUuuugacagcacu.......................................................................................................................................................................................   3       1   LT-2
......................ugCaucuuguuugacagcacu.......................................................................................................................................................................................   1       1   JCK-3
......................Cggaucuuguuugacagcacu.......................................................................................................................................................................................   1       1   JT-1
......................uggGucuuguuugacagcacu.......................................................................................................................................................................................   3       1   LT-3
......................uggaucuuguuugacagcacG.......................................................................................................................................................................................   16      1   JCK-2
......................uggCucuuguuugacagcacu.......................................................................................................................................................................................   95      1   LT-3
......................uggNucuuguuugacagcacu.......................................................................................................................................................................................   1       1   LCK-1
......................uUgaucuuguuugacagcacu.......................................................................................................................................................................................   2       1   LT-1
......................uggGucuuguuugacagcacu.......................................................................................................................................................................................   8       1   JCK-2
......................uggaucuuguuugacagcacu.......................................................................................................................................................................................   979     0   JT-3
......................uggaucuugAuugacagcacu.......................................................................................................................................................................................   1       1   JCK-2
......................uggCucuuguuugacagcacu.......................................................................................................................................................................................   39      1   LCK-3
......................uggaucuuguuugacagcaUu.......................................................................................................................................................................................   37      1   LCK-2
......................uggaucuugCuugacagcacu.......................................................................................................................................................................................   6       1   JCK-2
......................uggaucuuguuugacagcGcu.......................................................................................................................................................................................   2       1   JCK-1
......................uggGucuuguuugacagcacu.......................................................................................................................................................................................   1       1   JT-1
......................uggaucuuUuuugacagcacu.......................................................................................................................................................................................   4       1   JCK-2
```

Mature (uggaucuuguuugacagcacu) — **Star** (gugcugucgacaagaucuaga)

```
uccaucuuguucguuuacccucuggaucuuguuugacagcacucucuccuaucuuguuugacugaugaugaugggagugcugucgacaagaucuagaagguacugucgaauaagauacagauacggaggaaggaucaacuuuaccuacucucacacaaucuucuucauauucucuucaauuuuuauuuuuuuauuguucguauuuuuuuucccagauuagauuucccuguuaaucaccaugguauuaaca
......................uggaucuuguuugacagcacu.....   48441   0   JCK-3
......................uggaucuuguuugacagcaUu.....   1426    1   JCK-1
......................uggaucuuguuugacagcCcu.....   2       1   JT-1
......................uggaucuuguuugacagcacGc....   1       1   JCK-1
......................uggaucuuNuuugacagcacuc....   1       1   JCK-3
......................uggaucuugGuugacagcacuc....   3       1   JCK-2
......................uggGucuuguuugacagcacuc....   4       1   JCK-2
......................uUgaucuuguuugacagcacuc....   1       1   LT-2
......................uggaucuCguuugacagcacuc....   3       1   JCK-2
......................uAgaucuuguuugacagcacuc....   3       1   JCK-1
......................uggaucuuguuuAacagcacuc....   1       1   JCK-1
......................uggCucuuguuugacagcacuc....   3       1   LCK-3
......................uggaucuugCuugacagcacuc....   1       1   JCK-3
......................uggaucuuguuugacagcaAuc....   1       1   JCK-2
......................uggaucuuguuugaAagcacuc....   4       1   JCK-2
......................uggCucuuguuugacagcacuc....   4       1   LT-2
......................uggaucuuguuugacagcacuG....   7       1   LT-1
......................uggaucuuguuugNcagcacuc....   1       1   LCK-1
......................uNgaucuuguuugacagcacuc....   17      1   JCK-2
......................uggCucuuguuugacagcacuc....   7       1   LT-3
......................uggaucCuguuugacagcacuc....   2       1   JCK-2
......................uggaucuuguuugacagcCcuc....   1       1   JT-3
......................uggaucuuguuugacagcacuG....   3       1   LT-3
......................uggCucuuguuugacagcacuc....   1       1   JT-2
......................uggaucuuguuugacagcGcuc....   1       1   JCK-1
......................ugAaucuuguuugacagcacuc....   2       1   JCK-3
......................uggaucuuguuGgacagcacuc....   3       1   JCK-1
......................uggCucuuguuugacagcacuc....   36      1   JCK-2
......................uAgaucuuguuugacagcacuc....   1       1   LT-2
......................uggaucuuguuugaGagcacuc....   4       1   JCK-2
......................uggaucuuguuugacagcacuA....   38      1   JCK-1
......................uUgaucuuguuugacagcacuc....   1       1   JCK-2
......................uggaucuuguuugacagcacuA....   41      1   JCK-3
......................uggaucuuguuugacGgcacuc....   2       1   JCK-3
......................uggaucuuguuugacagcacuc....   17179   0   JCK-2
......................uggCucuuguuugacagcacuc....   33      1   JCK-3
......................uggaucuuguuugacagcacuc....   9941    0   JCK-1
......................uggaucuuguuugacagcacuA....   59      1   JCK-2
......................uggaucuuguuugacagcacuU....   48      1   LT-3
......................uggaucuuguuugacagcacuU....   64      1   LCK-3
......................uggaucuuguuugacaCcacuc....   1       1   JCK-3
......................uggaucuuguuugGcagcacuc....   3       1   JCK-2
......................uggaucuuguuugacagcacuc....   520     0   LCK-3
......................uggaucuuguuugacagcaUuc....   5       1   JCK-2
......................ugAaucuuguuugacagcacuc....   1       1   LT-1
......................ugAaucuuguuugacagcacuc....   1       1   LT-2
......................uggaucuuguuugacagcacuU....   1       1   JT-2
......................uggCucuuguuugacagcacuc....   2       1   LCK-1
......................uggCucuuguuugacagcacuc....   36      1   JCK-1
......................uggaCcuuguuugacagcacuc....   6       1   JCK-1
......................uggaucuuguuugacCgcacuc....   3       1   JCK-3
......................Cggaucuuguuugacagcacuc....   1       1   LCK-3
......................uggaucuuAuuugacagcacuc....   4       1   JCK-3
......................uggaucuuguuugacagcacuU....   22      1   LCK-2
......................uUgaucuuguuugacagcacuc....   1       1   JCK-3
......................uggGucuuguuugacagcacuc....   1       1   JCK-1
......................uggaucuuguuugacCgcacuc....   1       1   LT-2
......................uggaucuuguuugacagcaUuc....   7       1   JCK-1
......................uggaucuuguuuUacagcacuc....   1       1   JCK-1
......................uggaucuuguuugacagcacuN....   9       1   JCK-1
......................uggaucuuguuugacaAcacuc....   3       1   JCK-2
......................uggaucuuguuugacagcUcuc....   1       1   JCK-1
......................uggaucuCguuugacagcacuc....   2       1   JCK-1
......................uggauUuuguuugacagcacuc....   5       1   JCK-1
......................uggaucuuguuugacagUacuc....   5       1   JCK-2
......................uggaucuugGuugacagcacuc....   3       1   JCK-3
......................Cggaucuuguuugacagcacuc....   6       1   JCK-2
......................uggaucuuguuugCcagcacuc....   2       1   LT-3
......................uUgaucuuguuugacagcacuc....   1       1   JCK-1
......................Cggaucuuguuugacagcacuc....   1       1   LT-2
```

Mature **Star**

uccaucuuguucguuuacccucuggaucuuguuugacagcacucucuccuaucuuguuugacugaugaugauggagugcugucgacaagaucuagaagguacugucgaauaagauacagauacggaggaaggaucaacuuuaccuacucucacacaaucuucuucauauucucuucaauuuuuauuuuuuuauuguucguauuuuuuuucccagauuagauuucccuguuaaucaccaugguauuaaca

......................uggaucuuguuugCcagcacuc	2	1	JT-1
......................uggaucuuguuNgacagcacuc	29	1	JCK-1
......................uggaucuuguGugacagcacuc	5	1	JCK-1
......................uggaucuuguuugacagcacuG	1	1	LT-2
......................uggaucuuguuugacagcCcuc	7	1	JCK-2
......................uAgaucuuguuugacagcacuc	7	1	JCK-2
......................uNgaucuuguuugacagcacuc	15	1	JCK-3
......................uggaucCuguuugacagcacuc	1	1	JCK-1
......................Cggaucuuguuugacagcacuc	1	1	LT-1
......................uggaucuuguuugacagcCcuc	1	1	LCK-1
......................uggaucuuguuugCcagcacuc	44	1	JCK-2
......................uggaucuuguuugacagcacuG	2	1	JCK-3
......................uggaucuuguuugacagcaUuc	8	1	JCK-3
......................uggaucuuguuugacagcCcuc	9	1	JCK-3
......................uggaucuCguuugacagcacuc	2	1	JCK-3
......................uggaucuuUuuugacagcacuc	2	1	JCK-2
......................uggaucuuguuugacagcaUuc	3	1	LT-2
......................uggaucuuguuugacagcacuA	33	1	LT-2
......................uggaucuuguuugacagcacGc	1	1	JCK-2
......................uggaucuuguuugacagcacuG	11	1	JCK-1
......................uggaucuuguuugCcagcacuc	2	1	LT-2
......................ugUaucuuguuugacagcacuc	3	1	JCK-3
......................uggNucuuguuugacagcacuc	7	1	JCK-1
......................uggaGcuuguuugacagcacuc	1	1	JCK-3
......................uggaucuuguuugacCgcacuc	5	1	JCK-1
......................uggaucuuAuuugacagcacuc	1	1	LT-2
......................uggaucuuguuugacagNacuc	1	1	JCK-3
......................uggCucuuguuugacagcacuc	1	1	JT-1
......................uggaucuuguuugacagcacuU	68	1	JCK-1
......................uggaucuuguuuUacagcacuc	1	1	JCK-3
......................uggaucuuguuugCcagcacuc	24	1	JCK-1
......................uggaucuuguuugacagcacuA	12	1	LCK-3
......................uggaucuuguuugacaAcacuc	6	1	JCK-3
......................uggaucuugCuugacagcacuc	1	1	JCK-2
......................ugUaucuuguuugacagcacuc	1	1	JCK-2
......................uggaucuuguuugacagcacuc	220	0	JT-1
......................uAgaucuuguuugacagcacuc	1	1	LT-3
......................uggaucuuguuugCcagcacuc	1	1	JT-2
......................uggauUuuguuugacagcacuc	1	1	JCK-3
......................ugCaucuuguuugacagcacuc	1	1	JCK-2
......................uggaucuuguuugacagcacuU	85	1	JCK-3
......................uggaucuuguuugacagcacuU	4	1	JT-3
......................uNgaucuuguuugacagcacuc	26	1	JCK-1
......................uggaCcuuguuugacagcacuc	1	1	LT-2
......................uggaucuuguuugacagcacuU	97	1	JCK-2
......................uggaucuuguuugacagcacuA	11	1	JT-1
......................uggaucCuguuugacagcacuc	5	1	JCK-3
......................uggaucuuguuugacagcGcuc	3	1	JCK-2
......................uggaucuuguuugacagcUcuc	2	1	JCK-2
......................uggaucuuguuugacagcacuc	70	0	LCK-2
......................uggaucuuguuugCcagcacuc	7	1	LT-1
......................uggaucuuguuugacagcaNuc	11	1	JCK-1
......................uggaucuuguuugacagcNcuc	7	1	JCK-1
......................uggaucuuguuugacCgcacuc	2	1	LCK-3
......................uggaucuuguuugacaAcacuc	4	1	JCK-1
......................uggaucuuguuugacagcacuU	18	1	LCK-1
......................uggaucuuguuugacagcacuc	627	0	LT-2
......................uggaucuuguuugacagcacNc	3	1	JCK-1
......................uggaucuuguuugacagcacuc	138	0	JT-2
......................uggaucuuguuugacagUacuc	1	1	JCK-1
......................uggauAuuguuugacagcacuc	1	1	JCK-1
......................uggaucuuguuugCcagcacuc	19	1	JCK-3
......................uggaucuuguuugacagcCcuc	7	1	JCK-1
......................uggaucuuguuugacCgcacuc	1	1	LT-1
......................uggaucuuguuugacagcacuc	152	0	JT-3
......................uggaucuugGuugacagcacuc	3	1	JCK-1
......................uggauUuuguuugacagcacuc	7	1	JCK-2
......................uggaucuuguuugacagcacuA	32	1	LT-1
......................uggaucuuguuugacagcacGc	1	1	JCK-3
......................uggaucuuguuugacagcacuc	12872	0	JCK-3

```
                      Mature                                      Star
uccaucuuguucguuuacccucuggaucuuguuugacagcacucucuccuaucuuguuugacugaugaugaugggagugcugucgacaagaucuagaagguacugucgaauaagauacagauacggaggaaggaucaacuuuaccuacucucacacaaucuucuucauauucucuucaauuuuuauuuuuuuauuguucguauuuuuuucccagauuagauuucccuguuaaucaccaugguauuaaca
......................uggCucuuguuugacagcacuc................................................................................................................................................  4     1  LT-1
......................Cggaucuuguuugacagcacuc................................................................................................................................................  2     1  JCK-1
......................ugAaucuuguuugacagcacuc................................................................................................................................................  2     1  JCK-1
......................Cggaucuuguuugacagcacuc................................................................................................................................................  5     1  JCK-3
......................uggaucuuguuuAacagcacuc................................................................................................................................................  1     1  JCK-3
......................uggaucuuguuugCcagcacuc................................................................................................................................................  3     1  LCK-3
......................uggaucuuguuugacagcacuU................................................................................................................................................  27    1  LT-2
......................uggaucuuguuugacagcacuU................................................................................................................................................  47    1  LT-1
......................uggaucuuguuugacagcacuG................................................................................................................................................  4     1  JCK-2
......................uAgaucuuguuugacagcacuc................................................................................................................................................  2     1  LT-1
......................uggaucuuguuugacGgcacuc................................................................................................................................................  1     1  LT-1
......................uggaucuuguuugacagGacuc................................................................................................................................................  7     1  JCK-1
......................ugAaucuuguuugacagcacuc................................................................................................................................................  2     1  LT-3
......................uggaucuuUuuugacagcacuc................................................................................................................................................  1     1  JCK-3
......................uggaCcuuguuugacagcacuc................................................................................................................................................  9     1  JCK-3
......................ugAaucuuguuugacagcacuc................................................................................................................................................  4     1  JCK-2
......................uggaucuuguuugacagcacuc................................................................................................................................................  1069  0  LT-3
......................uAgaucuuguuugacagcacuc................................................................................................................................................  10    1  JCK-3
......................uggaucuuAuuugacagcacuc................................................................................................................................................  4     1  JCK-2
......................uggaucuuguuugacCgcacuc................................................................................................................................................  13    1  JCK-2
......................uggaucuuguuugacagcacuA................................................................................................................................................  34    1  LT-3
......................uAgaucuuguuugacagcacuc................................................................................................................................................  1     1  LCK-3
......................uggaucuuguuugacagcacuc................................................................................................................................................  76    0  LCK-1
......................ugUaucuuguuugacagcacuc................................................................................................................................................  1     1  JCK-1
......................uggaucuuguuuGcagcacuc.................................................................................................................................................  1     1  JCK-1
......................uggaucuuguuugacagcacuN................................................................................................................................................  4     1  LT-2
......................ugNaucuuguuugacagcacuc................................................................................................................................................  2     1  LT-2
......................uggaucuuguuuAacagcacuc................................................................................................................................................  3     1  JCK-2
......................uggaucuuguCugacagcacuc................................................................................................................................................  1     1  JCK-1
......................uggaucuuguuugacagcacuc................................................................................................................................................  1262  0  LT-1
......................uggGucuuguuugacagcacuc................................................................................................................................................  1     1  JCK-3
......................uggaucuuguuugacagcacucu...............................................................................................................................................  80    0  JCK-3
......................uggaucuuguuugacagcacuUu...............................................................................................................................................  112   1  JCK-3
......................uggaucuuguuugacagcacucA...............................................................................................................................................  10    1  JT-2
......................uggaucuuguuugacagcacuUu...............................................................................................................................................  159   1  JCK-2
......................uggaucuuguuNgacagcacucu...............................................................................................................................................  3     1  JCK-1
......................uggaucuuguuugacagcacucA...............................................................................................................................................  100   1  JCK-3
......................uggaucuuguuugacagcacuUu...............................................................................................................................................  23    1  LCK-1
......................uggaucuuguuugacagcacuGu...............................................................................................................................................  1     1  LT-3
......................uggaucuuguuugacagcacucu...............................................................................................................................................  10    0  LT-3
......................uggaucuuguuugacagcacucu...............................................................................................................................................  90    0  JCK-1
......................uggaucuuguuugacagcacuUu...............................................................................................................................................  39    1  JT-1
......................uggaucuuguuugacagcacuAu...............................................................................................................................................  3     1  JCK-1
......................uggaucuuguuugacagcacucA...............................................................................................................................................  24    1  LCK-3
......................uggaucuuguuugacagcacucu...............................................................................................................................................  16    0  LCK-3
......................uggaucuuguuugacagcacucG...............................................................................................................................................  4     1  JCK-3
......................uggaucuuguuugacagcacucA...............................................................................................................................................  140   1  JCK-2
......................uggaucuuguuugacagcacucG...............................................................................................................................................  5     1  JCK-2
......................uggaucuuguuugacagcacuAu...............................................................................................................................................  2     1  JCK-2
......................uggaucuuguuugacagcacuUu...............................................................................................................................................  62    1  LT-1
......................uggaucuuguuugacagcacucA...............................................................................................................................................  6     1  LT-3
......................uggaucuuguuugacagcacuUu...............................................................................................................................................  53    1  LT-3
......................uggaucuuguuugacagcacuGu...............................................................................................................................................  1     1  JCK-1
......................uggaucuuguuugacagcacuUu...............................................................................................................................................  75    1  LCK-3
......................uggaucuuguuugacagcacuUu...............................................................................................................................................  21    1  JT-3
......................uggCucuuguuugacagcacucu...............................................................................................................................................  1     1  LT-2
......................uggaucuuguuugacagcacuUu...............................................................................................................................................  109   1  JCK-1
......................uggaucuuguuugacagcacucA...............................................................................................................................................  14    1  LT-2
......................uggaucuuguuugacagcacuAu...............................................................................................................................................  1     1  JCK-3
......................uggaucuuguuugacagcacucu...............................................................................................................................................  26    0  LT-1
......................uggaucuuguuugacagcacuUu...............................................................................................................................................  19    1  LCK-2
......................uggaucuuguuugacagcacuGu...............................................................................................................................................  6     1  JCK-2
......................uggaucuuguuugacagcacucA...............................................................................................................................................  79    1  JCK-1
......................uggaucuuguuugacagcacuUu...............................................................................................................................................  21    1  JT-2
......................uggaucuuguuugacagcacuGu...............................................................................................................................................  2     1  LT-2
......................uggaucuuguuugacagcacuUu...............................................................................................................................................  71    1  LT-2
......................uggaucuuguuugacagcacucA...............................................................................................................................................  23    1  LT-1
......................uggaucuuguuugacagcacucG...............................................................................................................................................  3     1  JCK-1
......................uggaucuuguuugacagcacucu...............................................................................................................................................  14    0  LT-2
......................uggaucuuguuugacagcacucA...............................................................................................................................................  14    1  JT-3
```

Mature Star

```
uccaucuuguucguuuacccucuggaucuuguuugacagcacucucuccuaucuuguuugacugaugaugaugggagugcugucgacaagaucuagaagguacugucgaauaagauacagauacggaggaaggaucaacuuuaccuacucucacacaaucuucuucauauucucuucaauuuuuauuuuuuuauuguucguauuuuuuucccagauuagauuucccuguuaaucaccaugguauuaaca
......................uggaucuuguuugacagcacucu..........  111  0  JCK-2
......................uggaucuuguuugacagcacuGu..........  5    1  LT-1
......................uggaucuuguuugacagcacucAc.........  2    1  LT-3
......................uggaucuuguuugacagcacucuU.........  6    1  JCK-2
......................uggaucuuguuugacagcacucuU.........  1    1  JCK-1
......................uggaucuuguuugacagcacucuA.........  5    1  LT-2
......................uggaucuuguuugacagcacucuc.........  19   0  JCK-2
......................uggaucuuguuugacagcacucuc.........  19   0  JCK-3
......................uggaucuuguuugacagcacucuA.........  2    1  JCK-2
......................uggaucuuguuugacagcacucAc.........  13   1  LCK-3
......................uggaucuuguuugacagcacucuU.........  7    1  LT-2
......................uggaucuuguuugacagcacucAc.........  7    1  LT-1
......................uggaucuuguuugacagcacucuc.........  1    0  LT-3
......................uggaucuuguuNgacagcacucuc.........  1    1  JCK-1
......................uggaucuuguuugacagcacucuA.........  2    1  JCK-3
......................uggaucuuguuugacagcacuUuc.........  3    1  JCK-2
......................uggaucuuguuugacagcacuGuc.........  1    1  LT-3
......................uggaucuuguuugacagcacucuc.........  3    0  LCK-3
......................uggaucuuguuugacagcacucuc.........  12   0  JCK-1
......................uggaucuuguuugacagcacucuA.........  1    1  LT-1
......................uggaucuuguuugacagcacucuA.........  1    1  LT-3
......................uggaucuuguuugacagcacucuU.........  9    1  LT-1
......................uggaucuuguuugacagcacucuA.........  2    1  JCK-1
......................uggaucuuguuugacagcacucuc.........  1    0  LT-1
......................uggaucuuguuugacagcacucAcu........  1    1  JCK-2
......................uggaucuuguuugacagcacucuUu........  2    1  JCK-2
......................uggaucuuguuugacagcacucuUu........  6    1  LT-2
......................uggaucuuguuugacagcacucuUu........  1    1  JCK-1
......................uggaucuuguuugacagcacucuUu........  1    1  LT-1
......................uggaucuuguuugacagcacucucu........  1    0  LT-3
......................uggaucuuguuugacagcacucucu........  6    0  JCK-2
......................uggaucuuguuugacagcacucucu........  1    0  JCK-1
......................uggaucuuguuugacagcacucucu........  2    0  JCK-3
......................uggaucuuguuugacagcacucuAu........  1    1  LT-1
......................uggaucuuguuugacagcacucucGc.......  1    1  LT-1
......................uggaucuuguuugacagcacucuGuc.......  1    1  JCK-3
......................uggaucuuguuugacagcacucuGuc.......  2    1  JCK-2
......................uggaucuuguuugacagcacucucucc......  3    0  LCK-3
.......................ggaucuuguuugacagca..............  1    0  LT-1
.......................ggaucuuguuugacagca..............  1    0  JCK-1
.......................ggaucuuguuugacagca..............  2    0  JCK-3
.......................ggaucuuguuugacagcac.............  4    0  LCK-3
.......................ggaucuuguuugacagcac.............  1    0  LT-2
.......................ggaucuuguuugacagcac.............  2    0  LT-1
.......................ggaucuuguuugacagcacu............  5    0  LT-1
.......................ggaucuuguuugacagcacu............  5    0  LT-2
.......................ggaucuuguuugacagcacu............  2    0  LT-3
.......................ggaucuuguuugacagcacu............  4    0  JCK-3
.......................ggaucuuguuugacagcacu............  5    0  JCK-1
.......................ggaucuuguuugacagcacu............  3    0  JCK-2
.......................ggaucuuguuugacagcacu............  2    0  LCK-3
.......................ggaucuuguuugacagcacuc...........  6    0  JCK-1
.......................ggaucuuguuugacagcacuc...........  6    0  JCK-2
.......................ggaucuuguuugacagcacuc...........  14   0  JCK-3
.......................ggaucuuguuugacagcacucu..........  5    0  JCK-1
.......................ggaucuuguuugacagcacucA..........  2    1  JCK-2
.......................ggaucuuguuugacagcacucu..........  4    0  JCK-3
.......................ggaucuuguuugacagcacucu..........  6    0  JCK-2
.......................ggaucuuguuugacagcacucucu........  2    0  JCK-2
........................gaucuuguuugacagcacu............  14   0  LT-2
........................gaucuuguuugacagcacu............  3    0  LT-3
........................gaucuuguuugacagcacu............  1    0  LT-1
........................gaucuuguuugacagcacu............  1    0  JCK-2
........................gaucuuguuugacagcacu............  3    0  JCK-1
........................gaucuuguuugacagcacuc...........  3    0  LT-3
........................gaucuuguuugacagcacuc...........  2    0  LT-1
........................gaucuuguuugacagcacucA..........  1    1  LT-1
........................gaucuuguuugacagcacucu..........  1    0  LT-1
........................gaucuuguuugacagcacucuc.........  1    0  JCK-3
........................gaucuuguuugacagcacucuc.........  4    0  JCK-2
```

```
                      Mature                                               Star
uccaucuuguucguuuacccucuggaucuuguuugacagcacucucuccuaucuuguuugacugaugaugauggagugcugucgacaagaucuagaagguacugucgaauaagauacagauacggaggaaggaucaacuuuaccuacucucacacaaucuucuucauauucucuucaauuuuuauuuuuuuauuguucguauuuuuuuucccagauuagauuucccuguuaaucaccaugguauuaaca
........................gaucuuguuugacagcacucuc...........................................................................................................................................................................................................   1    0    JCK-1
.........................aucuuguuugacagcacu..............................................................................................................................................................................................................   3    0    LT-2
.........................aucuuguuugacagcacu..............................................................................................................................................................................................................   2    0    LT-1
.........................aucuuguuugacagcacuc.............................................................................................................................................................................................................   8    0    LCK-3
.........................aucuuguuugacagcacuc.............................................................................................................................................................................................................   5    0    LT-3
.........................aucuuguuugacagcacuc.............................................................................................................................................................................................................   1    0    JCK-2
.........................aucuuguuugacagcacuc.............................................................................................................................................................................................................   2    0    LT-1
.........................aucuuguuugacagcacuc.............................................................................................................................................................................................................   1    0    LT-2
.........................aucuuguuugacagcacuU.............................................................................................................................................................................................................   1    1    LT-2
.........................aucuuguuugacagcacuUu............................................................................................................................................................................................................   1    1    LT-1
.........................aucuuguuugacagcacucu............................................................................................................................................................................................................   4    0    LT-1
.........................aucuuguuugacagcacuUu............................................................................................................................................................................................................   1    1    LT-3
.........................aucuuguuugacagcacucA............................................................................................................................................................................................................   1    1    LT-2
.........................aucuuguuugacagcacucu............................................................................................................................................................................................................   4    0    LT-3
.........................aucuuguuugacagcacucA............................................................................................................................................................................................................   7    1    LCK-3
.........................aucuuguuugacagcacucA............................................................................................................................................................................................................   2    1    LT-1
.........................aucuuguuugacagcacucu............................................................................................................................................................................................................   24   0    LCK-3
.........................aucuuguuugacagcacucu............................................................................................................................................................................................................   2    0    LT-2
.........................aucuuguuugacagcacucu............................................................................................................................................................................................................   3    0    JCK-1
.........................aucuuguuugCcagcacucu............................................................................................................................................................................................................   1    1    JCK-2
.........................aucuuguuugacagcacucu............................................................................................................................................................................................................   15   0    JCK-2
.........................aucuuguuugacagcacucu............................................................................................................................................................................................................   4    0    JCK-3
.........................aucuuguuugacagcacucuc...........................................................................................................................................................................................................   11   0    JCK-3
.........................aucuuguuugacagcacucuc...........................................................................................................................................................................................................   8    0    JCK-1
.........................aucuuguuugacagcacucuA...........................................................................................................................................................................................................   1    1    JCK-3
.........................aucuuguuugacagcacucuc...........................................................................................................................................................................................................   12   0    JCK-2
.........................Cucuuguuugacagcacucuc...........................................................................................................................................................................................................   1    1    JCK-2
.........................aucuuguuugacagcacucuA...........................................................................................................................................................................................................   3    1    JCK-1
.........................aucuuguuugacagcacucuU...........................................................................................................................................................................................................   6    1    LCK-3
.........................aucuuguuugacagcacucuU...........................................................................................................................................................................................................   4    1    LT-2
.........................aucuuguuugacGgcacucucu..........................................................................................................................................................................................................   1    1    JCK-3
.........................aucuuguuugacagcacucCcu..........................................................................................................................................................................................................   1    1    LT-3
.........................Cucuuguuugacagcacucucu..........................................................................................................................................................................................................   1    1    JCK-1
.........................aucuuguuugacagcacucucG..........................................................................................................................................................................................................   1    1    JCK-1
.........................aucuuguuugacagcacucucu..........................................................................................................................................................................................................   107  0    JCK-2
.........................aucuuguuugacagcacucCcu..........................................................................................................................................................................................................   1    1    JCK-2
.........................aucuuguuugacagcacucucu..........................................................................................................................................................................................................   92   0    JCK-3
.........................aucuuguuugacagcacucCcu..........................................................................................................................................................................................................   1    1    JCK-3
.........................aucuuguuugacagcacucCcu..........................................................................................................................................................................................................   1    1    JCK-1
.........................aucuuguuugacagcacucucu..........................................................................................................................................................................................................   70   0    JCK-1
.........................aucuuguuugacagcacucucu..........................................................................................................................................................................................................   1    0    LT-1
.........................aucuuguuugCcagcacucucu..........................................................................................................................................................................................................   1    1    JCK-1
.........................aucuuguuugacagcacucucuA.........................................................................................................................................................................................................   1    1    JCK-1
.........................aucuuguuugacagcacucucuc.........................................................................................................................................................................................................   1    0    JCK-2
.........................aucuuguuugacagcacucucuA.........................................................................................................................................................................................................   1    1    JCK-2
.........................aucuuguuugacagcacucucuc.........................................................................................................................................................................................................   1    0    JCK-3
.........................aucuuguuugacagcacucucuc.........................................................................................................................................................................................................   1    0    JCK-1
.........................aucuuguuugacagcacucucucc........................................................................................................................................................................................................   1    0    LT-1
..........................ucuuguuugacagcacuc.............................................................................................................................................................................................................   1    0    LT-2
..........................ucuuguuugacagcacuc.............................................................................................................................................................................................................   2    0    LT-3
..........................ucuuguuugacagcacucu............................................................................................................................................................................................................   2    0    JT-2
..........................ucuuguuugacagcacucu............................................................................................................................................................................................................   2    0    JCK-2
..........................ucuuguuugacagcacucA............................................................................................................................................................................................................   1    1    LT-3
..........................ucuuguuugacagcacucu............................................................................................................................................................................................................   2    0    LT-1
..........................ucuuguuugacagcacucuc...........................................................................................................................................................................................................   2    0    JCK-2
..........................ucuuguuugacagcacucuc...........................................................................................................................................................................................................   1    0    JCK-3
..........................ucuuguuugacagcacucucu..........................................................................................................................................................................................................   6    0    JCK-3
..........................ucuuguuugacagcacucucu..........................................................................................................................................................................................................   13   0    JCK-2
..........................ucuuguuugacagcacucucu..........................................................................................................................................................................................................   3    0    JCK-1
..........................ucuuguuugacagcacucucu..........................................................................................................................................................................................................   1    0    LT-1
..........................ucuuguuugacagcacucucu..........................................................................................................................................................................................................   2    0    LT-3
..........................ucuuguuugacagcacucucuc.........................................................................................................................................................................................................   2    0    JCK-1
..........................ucuuguuugacagcacucucuc.........................................................................................................................................................................................................   1    0    LT-1
..........................ucuuguuugacagcacucucuc.........................................................................................................................................................................................................   4    0    JCK-2
..........................ucuuguuugacagcacucucuc.........................................................................................................................................................................................................   2    0    JCK-3
..........................ucuuguuugacagcacucucucA........................................................................................................................................................................................................   1    1    LT-1
...............................uuugacagcacucucuccu.......................................................................................................................................................................................................   2    0    LT-3
.......................................cacucucuccuaucuuguuu..............................................................................................................................................................................................   1    0    LT-3
............................................ucuccuaucuuguuugacugauga.....................................................................................................................................................................................   2    0    JCK-3
............................................ucuccuaucuuguuugacugaugaugaugg...............................................................................................................................................................................   1    0    LT-3
```

Mature (uggaucuuguuugacagcacu) **Star** (gugcugucgacaagaucuaga)

uccaucuuguucguuuacccucuggaucuuguuugacagcacucucuccuaucuuguuugacugaugaugaugggagugcugucgacaagaucuagaagguacugucgaauaagauacagauacggaggaaggaucaacuuuaccuacucucacacaaucuucuucauauucucuucaauuuuuauuuuuuuauuguucguauuuuuuuucccagauuagauuucccuguuaaucaccaugguauuaaca

ccuaucuuguuugacugaugaug	1	0	LT-1
cuaucuuguuugacugaugau	1	0	LT-1
ucuuguuugacugaugaugau	2	0	LT-2
uuguuugacugaugaugaugggag	6	0	LCK-3
ugacugaugaugaugggagugcug	1	0	LT-3
cugaugaugaugggagugcugucgacaaga	2	0	LCK-2
cugaugaugaugggagugcugucgacaaga	5	0	LCK-1
gaugaugaugggagugcugu	1	0	JCK-1
gaugaugaugggagugcugu	1	0	JCK-2
gaugaugaugggagugcugucgacaagauc	1	0	LT-2
ugaugaugggagugcugucgac	1	0	LT-1
gaugaugggagugcugucgacaa	7	0	LCK-3
gaugaugggagugcugucgacaagauc	1	0	LT-1
augaugggagugcugucgaca	2	0	JCK-2
ugaugAgagugcugucgacaagauc	1	1	JCK-1
ugaugAgagugcugucgacaagauc	2	1	JCK-2
ugaugAgagugcugucgacaagauc	1	1	JCK-3
gaugggagugcugucgacaaga	1	0	LT-3
augggagugcugucgacaagauc	1	0	LT-2
augggagugcugucgacaagauc	8	0	LCK-3
augggagugcugucgacaagauc	1	0	JCK-2
augggagugcugucgacaagauc	1	0	JCK-1
ugggagugcugucgacaagauc	1	0	JCK-3
ugggagugcugucgacaagauc	4	0	LT-1
ugggagugcugucgacaagauc	1	0	LT-3
ugAgagugcugucgacaagauc	1	1	JCK-1
ugggagugcugucgacaagauc	6	0	JCK-2
ugAgagugcugucgacaagauc	1	1	JCK-2
ugggagugcugucgacaagaucu	1	0	JCK-3
gggagugcugucgacaagauc	3	0	LT-1
gggagugcugucgacaagauc	4	0	JCK-1
gggagugcugucgacaagauc	8	0	LT-3
gggagugcugucgacaagauc	1	0	JCK-3
gAgagugcugucgacaagauc	2	1	JCK-1
gggagugcugucgacaagauc	6	0	LT-2
gggagugcugucgacaagauc	3	0	JCK-2
gggagugcugucgacaagauc	19	0	LCK-2
gggagugcugucgacaagauc	1	0	LCK-3
gggagugcugucgacaagauc	26	0	LCK-1
Agagugcugucgacaagau	1	1	JCK-1
Agagugcugucgacaagau	1	1	JCK-3
Agagugcugucgacaagau	22	1	JT-1
Agagugcugucgacaagau	2	1	JCK-2
Agagugcugucgacaagauc	70	1	JCK-2
Agagugcugucgacaagauc	10	1	JT-1
Agagugcugucgacaagauc	55	1	JCK-3
Agagugcugucgacaagauc	51	1	JCK-1
ggagugcugucgacaagaucu	1	0	JCK-1
ggagugcugucgacaagaucu	2	0	JCK-3
ggagugcugucgacaagaucuag	2	0	JCK-3
ggagugcugucgacaagaucuagU	1	1	JCK-1
ggagugcugucgacaagaucuaga	1	0	LT-3
ggagugcugucgacaagaucuagC	3	1	LT-1
ggagugcugucgacaagaucuagU	1	1	LT-3
gagugcugucgacaagauc	8	0	JCK-2
gagugcugucgacaagauc	2	0	LT-3
gagugcugucgacaagauc	3	0	JCK-3
gagugcugucgacaagauc	3	0	LT-2
gagugcugucgacaagauc	9	0	JCK-1
gagugcugucgacaagaucC	2	1	JCK-1
gagugcugucgacaagaucC	8	1	JT-3
gagugcugucgacaagaucC	9	1	JCK-2
gagugcugucgacaagaucC	5	1	JT-2
gagugcugucgacaUgaucu	1	1	LT-1
gagugcugucgacaagaucu	2	0	LT-3
gagugcugucgacaagaucC	10	1	JCK-3
gagugcugucgacaagaucuG	1	1	LT-1
gagugcugucgacaagaucCa	15	1	JCK-2
gagugcugucgacaagaucCa	8	1	JCK-1
gagugcugucgacaagaucCa	8	1	JCK-3

```
                Mature                                                 Star
uccaucuuguucguuuacccucuggaucuuguuugacagcacucucuccuaucuuguuugacugaugaugaugggagugcugucgacaagaucuagaagguacugucgaauaagauacagauacggaggaaggaucaacuuuaccuacucucacacaaucuucuucauauucucuucaauuuuuauuuuuuuauuguucguauuuuuuuucccagauuagauuucccuguuaaucaccaugguauuaaca
..........................................................................gagugcugucgacaagaucuag..........................................................................................................................................................	2	0	JCK-2
..........................................................................gagugcugucgacaagaucuag..........................................................................................................................................................	2	0	LT-3
..........................................................................gagugcugucgacaagaucuagU.........................................................................................................................................................	1	1	JCK-2
..........................................................................gagugcugucgacaagaucCaga.........................................................................................................................................................	72	1	JCK-2
..........................................................................gagugcugucgNcaagaucuaga.........................................................................................................................................................	1	1	JCK-1
..........................................................................gagugcugucgacaagaucuagU.........................................................................................................................................................	1	1	JCK-3
..........................................................................gagugcugucgacaagaucuaga.........................................................................................................................................................	15	0	JCK-2
..........................................................................gagugcugucgacaagaucCaga.........................................................................................................................................................	35	1	JCK-1
..........................................................................gagugcugucgacaagaucCaga.........................................................................................................................................................	44	1	JCK-3
..........................................................................gagugcugucgacaagaucuaga.........................................................................................................................................................	9	0	LT-1
..........................................................................gagugcugucgacaagaucuaga.........................................................................................................................................................	6	0	JCK-1
..........................................................................gagugcugucgacaagaucuaga.........................................................................................................................................................	7	0	JCK-3
..........................................................................gagugcugucgacaagaucuaga.........................................................................................................................................................	1	0	LT-3
..........................................................................gagugcugucgacaagaucuagaagg......................................................................................................................................................	1	0	LT-3
..........................................................................gagugcugucgacaagaucuagaagg......................................................................................................................................................	5	0	LCK-3
...........................................................................agugcugucgacaagauA.............................................................................................................................................................	1	1	JCK-3
...........................................................................agugcugucgacaagauc.............................................................................................................................................................	3	0	JCK-2
...........................................................................agugcugucgacaagauc.............................................................................................................................................................	2	0	LCK-3
...........................................................................agugcugucgacaagauc.............................................................................................................................................................	2	0	LT-3
...........................................................................agugcugucgacaagauc.............................................................................................................................................................	1	0	JCK-3
...........................................................................agugcugucgacaagauc.............................................................................................................................................................	1	0	LT-1
...........................................................................agugcugucgacaagaucu............................................................................................................................................................	2	0	JCK-3
...........................................................................agugcugucgacaagaucC............................................................................................................................................................	7	1	JCK-2
...........................................................................agugcugucgacaaAaucu............................................................................................................................................................	2	1	LCK-3
...........................................................................agugcugucgacaagaucC............................................................................................................................................................	3	1	JCK-3
...........................................................................agugcugucgacaagaucu............................................................................................................................................................	3	0	LCK-3
...........................................................................agugcugucgacaagaucua...........................................................................................................................................................	1	0	JCK-3
...........................................................................agugcugucgacaagaucCa...........................................................................................................................................................	9	1	JCK-2
...........................................................................agugcugucgacaagaucCa...........................................................................................................................................................	4	1	JCK-3
...........................................................................agugcugucgacaagaucCa...........................................................................................................................................................	2	1	JCK-1
...........................................................................agugcugucgacaagaucua...........................................................................................................................................................	2	0	LT-3
...........................................................................agugcugucgacaagaucua...........................................................................................................................................................	1	0	LT-1
...........................................................................agugcugucgacaagaucuag..........................................................................................................................................................	2	0	LT-3
...........................................................................agugcugucgacaagaucCag..........................................................................................................................................................	4	1	JCK-3
...........................................................................agugcugucgacaagaucCag..........................................................................................................................................................	2	1	JCK-2
...........................................................................agugcugucgacaagaucuag..........................................................................................................................................................	2	0	JCK-3
...........................................................................agugcugucgacaagaucCag..........................................................................................................................................................	5	1	JCK-1
...........................................................................agugcugucgacaagaucuag..........................................................................................................................................................	3	0	JCK-2
...........................................................................agugcugucgacaagaucuag..........................................................................................................................................................	10	0	LT-2
...........................................................................agugcugucgacaagaucuaga.........................................................................................................................................................	1	0	LT-3
...........................................................................agugcugucgacaagaucuagU.........................................................................................................................................................	1	1	JCK-1
...........................................................................agugcugucgacaagaucuagU.........................................................................................................................................................	1	1	JCK-3
...........................................................................agugcugucgacaagaucuagU.........................................................................................................................................................	10	1	LCK-3
...........................................................................agugcugucgacaagaucCaga.........................................................................................................................................................	5	1	JCK-1
...........................................................................agugcugucgacaagaucCaga.........................................................................................................................................................	8	1	JCK-2
...........................................................................agugcugucgacaagaucCaga.........................................................................................................................................................	1	1	JCK-3
...........................................................................agugcugucgacaagaucCagaa........................................................................................................................................................	1	1	JCK-2
...........................................................................agugcugucgacaagaucuagaag.......................................................................................................................................................	1	0	JCK-2
...........................................................................agugcugucgacaagaucuagaag.......................................................................................................................................................	3	0	JCK-3
............................................................................gugcugucgacaagaucu............................................................................................................................................................	25	0	JCK-3
............................................................................gugcugucgacaagaucC............................................................................................................................................................	16	1	JCK-1
............................................................................gugcugucgacaagaucC............................................................................................................................................................	42	1	JCK-2
............................................................................gugcugucgacaagauUu............................................................................................................................................................	2	1	JCK-1
............................................................................gugcugucgacaagaucu............................................................................................................................................................	1	0	LCK-2
............................................................................gugcugucgacaagaucC............................................................................................................................................................	5	1	JT-3
............................................................................gugcugucgacaagaucu............................................................................................................................................................	2	0	JT-3
............................................................................gugcugucgacaagaucu............................................................................................................................................................	21	0	LT-1
............................................................................gugcugucgacaagaucu............................................................................................................................................................	24	0	JCK-2
............................................................................gugcugucgacaagaucu............................................................................................................................................................	14	0	LT-2
............................................................................gugcugucgacaagaucu............................................................................................................................................................	36	0	LCK-3
............................................................................gugcugucgacaagaucu............................................................................................................................................................	27	0	JCK-1
............................................................................Cugcugucgacaagaucu............................................................................................................................................................	1	1	LCK-3
............................................................................gugcugucgacaagaucC............................................................................................................................................................	18	1	JCK-3
............................................................................gugcugucgacCagaucu............................................................................................................................................................	1	1	LCK-2
............................................................................gugcugucgacaagaucC............................................................................................................................................................	3	1	JT-2
............................................................................gugcugucgacaagaucu............................................................................................................................................................	29	0	LT-3
............................................................................gugcugucgacaagaucu............................................................................................................................................................	1	0	JT-1
............................................................................gugcugucgacaagaucu............................................................................................................................................................	1	0	LCK-1
............................................................................gugcugucgacaagaucua...........................................................................................................................................................	4	0	JCK-2
............................................................................gugcugucgacaagaucua...........................................................................................................................................................	23	0	LT-2
```

```
                      Mature                                                Star
uccaucuuguucguuuacccucuggaucuuguuugacagcacucucuccuaucuuguuugacugaugaugaugggagugcugucgacaagaucuagaagguacugucgaauaagauacagauacggaggaaggaucaacuuuaccuacucucacacaaucuucuucauauucucuucaauuuuuauuuuuuuauuguucguauuuuuuuucccagauuagauuucccuguuaaucaccaugguauuaaca
............................................................................gugcugucgacaagaucCa...........................................................................................................................................................  11    1    JCK-1
............................................................................Augcugucgacaagaucua...........................................................................................................................................................  1     1    LT-2
............................................................................gugcugucgacaagaucCa...........................................................................................................................................................  9     1    JCK-3
............................................................................gugcugucgacaagaucCa...........................................................................................................................................................  27    1    JCK-2
............................................................................gugcugucgacaagaucCa...........................................................................................................................................................  8     1    JT-3
............................................................................gugcugucgacaaNaucua...........................................................................................................................................................  1     1    LCK-1
............................................................................gugcugucgacaagaucua...........................................................................................................................................................  7     0    JCK-1
............................................................................gugcugucgCcaagaucua...........................................................................................................................................................  1     1    LT-2
............................................................................gugcugucgacaagaucua...........................................................................................................................................................  36    0    LCK-3
............................................................................gugcugucgacaagaucua...........................................................................................................................................................  10    0    LT-1
............................................................................gugcugucgacaagaucua...........................................................................................................................................................  23    0    LCK-1
............................................................................gugcugucgacaagaucCa...........................................................................................................................................................  7     1    JT-1
............................................................................gugcugucgacaagaucua...........................................................................................................................................................  2     0    JCK-3
............................................................................gugcugucgacaagaucua...........................................................................................................................................................  25    0    LT-3
............................................................................gugcugucgacaagaucuU...........................................................................................................................................................  10    1    LCK-3
............................................................................gugcugucgacaagaucua...........................................................................................................................................................  14    0    LCK-2
............................................................................gugcugucgacaagaucCa...........................................................................................................................................................  7     1    JT-2
............................................................................gugcugucgacaagaucuag..........................................................................................................................................................  33    0    JCK-2
............................................................................gugcugucgacaagaucCag..........................................................................................................................................................  32    1    JCK-2
............................................................................gugcugucgacaagaucuaA..........................................................................................................................................................  1     1    LT-1
............................................................................gugcugucgacaagaucuag..........................................................................................................................................................  11    0    LT-1
............................................................................gugcugucgacaagaucCag..........................................................................................................................................................  9     1    JT-3
............................................................................gugcugucgacaagaucuag..........................................................................................................................................................  39    0    LT-3
............................................................................gugcugucgacaagaucuag..........................................................................................................................................................  14    0    JCK-3
............................................................................gugcugucgacaagaCcuag..........................................................................................................................................................  1     1    LT-3
............................................................................gugcugucgacaagaucuag..........................................................................................................................................................  15    0    LCK-3
............................................................................gugcugucgacaagaucCag..........................................................................................................................................................  7     1    JCK-1
............................................................................gugcugucgacaagaucCag..........................................................................................................................................................  11    1    JCK-3
............................................................................gugcugucgacaagaucCag..........................................................................................................................................................  9     1    JT-2
............................................................................gugcugucgacaagaucuag..........................................................................................................................................................  24    0    JCK-1
............................................................................gugcugucgacaagaucuag..........................................................................................................................................................  23    0    LT-2
............................................................................guUcugucgacaagaucuag..........................................................................................................................................................  1     1    LT-3
............................................................................gugcugucgacNagaucuaga.........................................................................................................................................................  1     1    JCK-1
............................................................................gugcugucgacaagaucuaga.........................................................................................................................................................  238   0    LT-3
............................................................................gugcugucgacaagCucuaga.........................................................................................................................................................  1     1    JCK-3
............................................................................gugcugucgacaUgaucuaga.........................................................................................................................................................  4     1    JCK-2
............................................................................gugcugucgacaagaucCaga.........................................................................................................................................................  910   1    JCK-3
............................................................................gugcugucgacaagGucuaga.........................................................................................................................................................  1     1    LCK-3
............................................................................gugcugucgacaCgaucuaga.........................................................................................................................................................  1     1    LT-3
............................................................................gugcugucgacaGgaucuaga.........................................................................................................................................................  1     1    LT-3
............................................................................gugcugucgacaagaucuaga.........................................................................................................................................................  191   0    JCK-1
............................................................................gugcugucgacaagaucuagG.........................................................................................................................................................  1     1    JCK-2
............................................................................gugUugucgacaagaucuaga.........................................................................................................................................................  2     1    JCK-1
............................................................................gugcugucgacaaAaucuaga.........................................................................................................................................................  2     1    JCK-2
............................................................................gugcugucgacaagaucCaga.........................................................................................................................................................  712   1    JCK-1
............................................................................gugcugucgacaagaucuaga.........................................................................................................................................................  9     0    JT-3
............................................................................gugcugucgacaagaucuagU.........................................................................................................................................................  7     1    JCK-2
............................................................................gugcugucgacaagaucuaga.........................................................................................................................................................  167   0    LCK-1
............................................................................gugcugucgacaUgaucuaga.........................................................................................................................................................  2     1    JCK-1
............................................................................gugcugucgCcaagaucuaga.........................................................................................................................................................  3     1    JCK-1
............................................................................gugUugucgacaagaucuaga.........................................................................................................................................................  1     1    LT-3
............................................................................gugcugucgacaagaucuaga.........................................................................................................................................................  5     0    JT-2
............................................................................gugcugucgacaUgaucuaga.........................................................................................................................................................  1     1    JCK-3
............................................................................gugcugucgacaagaucCaga.........................................................................................................................................................  18    1    JT-2
............................................................................Uugcugucgacaagaucuaga.........................................................................................................................................................  1     1    JCK-2
............................................................................gugcugucgacaaAaucuaga.........................................................................................................................................................  1     1    JCK-3
............................................................................gugcugucgCcaagaucuaga.........................................................................................................................................................  1     1    JCK-2
............................................................................gugcugucgacaagaucuaga.........................................................................................................................................................  169   0    LT-1
............................................................................gugcugucgacaagaucuagU.........................................................................................................................................................  4     1    JCK-3
............................................................................gugcugucgacaagaucuNga.........................................................................................................................................................  1     1    JCK-1
............................................................................gugcugucgacaagaucuagU.........................................................................................................................................................  8     1    LT-3
............................................................................gugcugucgacaagaucuagU.........................................................................................................................................................  3     1    JCK-1
............................................................................gugUugucgacaagaucuaga.........................................................................................................................................................  1     1    JCK-3
............................................................................gugcugucgacaagaucuaga.........................................................................................................................................................  113   0    LCK-3
............................................................................gugcCgucgacaagaucuaga.........................................................................................................................................................  1     1    LCK-1
............................................................................gugcugucgacaCgaucuaga.........................................................................................................................................................  1     1    LCK-2
............................................................................gugcugucgacaagaucCaga.........................................................................................................................................................  22    1    JT-3
............................................................................Augcugucgacaagaucuaga.........................................................................................................................................................  2     1    JCK-2
............................................................................gugcugucgacaagaucGaga.........................................................................................................................................................  1     1    JCK-1
............................................................................gugcugucgacaagaucCaga.........................................................................................................................................................  1129  1    JCK-2
```

Mature uggaucuuguuugacagcacu — **Star** gugcugucgacaagaucuaga

uccaucuuguucguuuacccucuggaucuuguuugacagcacucucuccuaucuuguuugacugaugaugaugggagugcugucgacaagaucuagaagguacugucgaauaagauacagauacggaggaaggaucaacuuuaccuacucucacacaaucuucuucauauucucuucaauuuuuauuuuuuuauuguucguauuuuuuuucccagauuagauuucccuguuaaucaccaugguauuaaca

gugcugucgacaagaucuaga	348	0	JCK-2
gugcugucgacaagaucuaga	265	0	JCK-3
gugcugucgacaagaucuaga	158	0	LCK-2
gugcugucgacaagaucuaga	153	0	LT-2
gugcugucgacaagaucuagU	5	1	LT-1
gugcugucgacaagaucCaga	19	1	JT-1
guNcugucgacaagaucuaga	1	1	LCK-1
gugcugucgCcaagaucuaga	1	1	LCK-2
gugcugucgacaagCucuaga	1	1	JCK-1
gugcugucgacaagaucuagaU	2	1	JCK-3
gugcugucgacaagaucuagaa	2	0	LT-1
gugcugucgacaagaucuagaU	1	1	JCK-1
gugcugucgacaagaucCagaa	7	1	JCK-2
gugcugucgacaagaucuagaa	5	0	LT-2
gugcugucgacaagaucCagaa	6	1	JCK-1
gugcugucgacaagaucCagaa	2	1	JCK-3
gugcugucgacaagaucuagaaA	3	1	LCK-3
gugcugucgacaagaucuagaag	1	0	JCK-3
gugcugucgacaagaucuagaaA	2	1	LT-3
gugcugucgacaagaucuagaag	1	0	JCK-2
gugcugucgacaagaucuagaag	2	0	JCK-1
gugcugucgacaagaucuagaagg	4	0	LCK-3
gugcugucgacaagaucuagaagg	1	0	LT-1
ugcugucgacaagaucua	1	0	JCK-1
ugcugucgacaagaucua	4	0	LT-1
ugcugucgacaagaucua	1	0	JCK-2
ugcugucgacaagaucua	12	0	LT-2
ugcugucgacaagaucua	19	0	LT-3
ugcugucgacaagaucua	31	0	LCK-3
ugcugucgacaagaucCag	2	1	JCK-3
ugcugucgacaagaucuag	12	0	LCK-3
ugcugucgacaagaucuag	9	0	LT-3
ugcugucgacaagaucuag	2	0	JCK-1
ugcugucgacaagaucuag	1	0	JT-2
ugcugucgacaagaucuag	3	0	JT-3
ugcugucgacaagaucCag	2	1	JCK-1
ugcugucgacaagaucuag	1	0	JCK-2
ugcugucgacaagaucuaA	1	1	LT-3
ugcugucgacaagaucuag	1	0	JCK-3
ugcugucgacaagaucuaA	1	1	JCK-1
ugcugucgacaagaucuag	14	0	LT-1
ugcugucgacaagaucuag	2	0	LT-2
ugcugucgacaagaucCag	2	1	JCK-2
ugcugucgacaUgaucuaga	1	1	JCK-3
ugcugucgacaagaucCaga	125	1	JCK-2
ugcugucgacaagaucuaga	69	0	JCK-3
uAcugucgacaagaucuaga	2	1	LT-2
ugcugucgacaagaucuCga	1	1	JT-2
ugcugucgCcaagaucuaga	1	1	LT-3
ugcugucgacaagaucuaga	20	0	JT-3
ugcugucgCcaagaucuaga	1	1	LT-2
ugcugucgacaagaucuCga	2	1	LT-2
ugcugucgacCagaucuaga	1	1	LT-1
ugcugucgCcaagaucuaga	1	1	JCK-3
uAcugucgacaagaucuaga	1	1	LT-3
ugcugucgacaagaucuaga	150	0	LT-2
ugcugucgacaagaucuaga	51	0	JCK-1
ugcugucgacaagaucCaga	105	1	JCK-3
ugcugucgacaagaucuaga	107	0	JCK-2
ugcugucgacaagaucCaga	13	1	JT-2
ugcugucgacaagaucuaga	127	0	LT-1
ugcugucgacCagaucuaga	1	1	LT-3
ugNugucgacaagaucuaga	2	1	LT-2
ugcugucgacaagaucuaga	94	0	LCK-3
ugcugucgacaagaucCaga	79	1	JCK-1
ugcugucgaAaagaucuaga	1	1	LT-2
ugcugucgacaagaucCaga	20	1	JT-3
ugcugucgacaagaucuaga	13	0	JT-1
ugcugucgacaagaucCaga	22	1	JT-1
ugcugucgacaagaucuaga	156	0	LT-3

Mature **Star**

uccaucuuguucguuuacccucuggaucuuguuugacagcacucucuccuaucuuguuugacugaugaugaugggagugcugucgacaagaucuagaagguacugucgaauaagauacagauacggaggaaggaucaacuuuaccuacucucacacaaucuucuucauauucucuucaauuuuuauuuuuuuauuguucguauuuuuuuucccagauuagauuucccuguuaaucaccaugguauuaaca

ugcugucgacaagaucuaga	13	0	JT-2
ugcugucgacaagaucuCga	1	1	LT-3
ugcugucgacaagCucuaga	1	1	LT-1
ugcugucgacaagaucuagU	7	1	LT-3
ugcugucgacaagCucuaga	1	1	JT-3
ugcugucgacaagaucuagaU	7	1	LT-1
ugcugucgacaagaucuagaa	1	0	LT-3
ugcugucgacaagaucCagaa	1	1	JCK-3
ugcugucgacaagaucuagaU	6	1	LT-2
ugcugucgacaagaucuagaa	3	0	JCK-2
ugcugucgacaagaucuagaU	3	1	JCK-3
ugcugucgacaagaucuagaa	3	0	LT-1
ugcugucgacaagaucuagaa	1	0	LT-2
ugcugucgacaagaucCagaa	2	1	JCK-1
ugcugucgacaagaucuagaa	1	0	JCK-1
ugcugucgacaagaucuagaU	3	1	LT-3
ugcugucgacaagaucuagaU	1	1	JCK-2
ugcugucgacaagaucuagaagg	2	0	JCK-2
ugcugucgacaagaucuagaagg	5	0	LT-3
ugcugucgacaagaucuagaagg	1	0	JCK-3
ugcugucgacaagaucCagaaggu	1	1	JCK-2
gcugucgacaagaucuag	1	0	JCK-2
gcugucgacaagaucuag	2	0	LT-2
gcugucgacaagaucuaga	6	0	LT-1
gcugucgacaagaucuaga	14	0	LT-3
gcugucgacaagaucuaga	3	0	JCK-3
gcugucgacaagaucuaga	37	0	LCK-2
gcugucgacaagaucuaga	3	0	JCK-1
gcugucgacaagaucuaga	1	0	JT-2
gcugucgacaagaucuaga	4	0	LCK-3
gcugucgacaagaucuaga	4	0	LT-2
Ucugucgacaagaucuaga	1	1	JT-2
gcugucgacaagaucuaga	3	0	JT-3
gcugucgacaagaucuaga	8	0	JCK-2
gcugucgacaagaucuaga	32	0	LCK-1
gcugucgacaagaucuagaa	1	0	JCK-2
gcugucgacaagaucuagaa	1	0	JCK-3
gcugucgacaagaucuagaa	5	0	LT-1
gcugucgacaagaucuagaa	3	0	JT-1
gcugucgacaagaucuagaa	2	0	LT-3
gcugucgacaagaucuagaag	21	0	JCK-2
gcugucgacaagaucuagaag	17	0	LT-3
gcugucgacaagaucuagaag	12	0	LT-2
Ucugucgacaagaucuagaag	1	1	LT-3
gcugucgacaagaucuagaag	5	0	LCK-3
gcugucgacaagaucCagaag	3	1	JCK-1
gcugucgacaagaucuagaag	11	0	JT-2
Acugucgacaagaucuagaag	1	1	LT-1
gcugucgacaagaucuagaag	18	0	JCK-3
gcugucgacaagaucuagaag	9	0	LT-1
gcugucgacaagaucCagaag	3	1	JT-2
gcugucgacaagaucCagaag	3	1	JCK-3
gcugucgacaagaucuagaag	2	0	JT-3
gcugucgacaagaucCagaag	1	1	JCK-2
gcugucgacaagaucCagaag	4	1	JT-3
gcugucgacaagaucuagaag	25	0	JCK-1
gcugucgacaagaucuagaagAu	1	1	LT-1
gcugucgacaagaucuagaaggu	1	0	LT-2
cugucgacaagaucuaga	1	0	JCK-1
cugucgacaagaucuaga	6	0	LT-3
cugucgacaagaucuaga	8	0	LCK-3
cugucgacaagaucuaga	5	0	JT-3
cugucgacaagaucCaga	1	1	JCK-2
cugucgacaagaucuaga	12	0	LT-2
cugucgacaagaucuaga	4	0	JT-2
cugucgacaagaucuaga	1	0	LT-1
cugucgacaagaucCaga	1	1	JCK-1
cugucgacaagaucuagaa	3	0	LT-2
cugucgacaagaucuagaa	9	0	JT-2
cugucgacaagaucuagaa	2	0	JCK-3

```
                                        Mature                              Star
uccaucuuguucguuuacccucuggaucuuguuugacagcacucucuccuaucuuguuugacugaugaugaugggagugcugucgacaagaucuagaagguacugucgaauaagauacagauacggaggaaggaucaacuuuaccuacucucacacaaucuucuucauauucucuucaauuuuuauuuuuuuauuguucguauuuuuuuucccagauuagauuucccuguuaaucaccaugguauuaaca
...............................................................................cugucgacaagaucCagaa   2   1   JCK-1
...............................................................................cugucgacaagaucuagaa   3   0   LT-1
...............................................................................cugucgacaagaucuagaa   1   0   JCK-2
...............................................................................cugucgacaagaucCagaa   1   1   JCK-3
...............................................................................cugucgacaagaucCagaa   2   1   JCK-2
...............................................................................cugucgacaagaucuagaa   6   0   JT-3
...............................................................................cugucgacaagaucuagaag   18   0   LT-2
...............................................................................cugucgacaagaucuagaaU   2   1   LT-1
...............................................................................cugucgacaagaucuagaag   3   0   JCK-1
...............................................................................cugucgacaagaucCagaag   5   1   JCK-3
...............................................................................cugucgacaagaucCagaag   1   1   JCK-1
...............................................................................cugucgCcaagaucuagaag   1   1   LT-1
...............................................................................cugucgacaagaucuagaaU   1   1   JCK-3
...............................................................................cugucgCcaagaucuagaag   1   1   LT-2
...............................................................................cugucgacaagaucuagaag   6   0   LT-1
...............................................................................cugucgacaagaucuagaag   3   0   JCK-3
...............................................................................cugucgacaagaucuagaaC   1   1   JCK-2
...............................................................................cugucgacaagaucCagaag   4   1   JCK-2
...............................................................................cugucgacaagaucuagaag   7   0   LT-3
...............................................................................cugucgacaagaucCagaagg   3   1   JCK-3
...............................................................................cugucgacaagaucCagaagg   5   1   JCK-1
...............................................................................cugucgacaagaucuagaagg   16   0   LT-3
...............................................................................cugucgacaagaucuagaagg   4   0   JCK-2
...............................................................................cugucgacaagaucuagaagg   7   0   JCK-3
...............................................................................cugucgacaagaucuagaagU   1   1   LT-3
...............................................................................cugucgacaagaucuagaagg   1   0   JCK-1
...............................................................................cugucgacaagaucuagaagA   3   1   LCK-3
...............................................................................cugucgacaagaucuagaagU   2   1   LT-2
...............................................................................cugucgacaagaucCagaagg   4   1   JCK-2
...............................................................................cugucgacaagaucuagaagU   1   1   LT-1
...............................................................................cugucgacaagaucuagaagg   22   0   LCK-2
...............................................................................cugucgacaagaucuagaagg   14   0   LT-2
...............................................................................cugucgacaagaucuagaagg   1   0   LT-1
...............................................................................cugucgacaagaucuagaagg   12   0   LCK-1
...............................................................................cugucgacaagaucuagaaggu   2   0   LT-1
...............................................................................cugucgacaagaucuagaagUu   4   1   LCK-3
...............................................................................cugucgacaagaucuagaagAu   2   1   LT-2
...............................................................................cugucgacaagaucuagaagguU   10   1   LCK-3
...............................................................................cugucgacaagaucuagaaggua   1   0   LT-3
................................................................................ugucgacaagaucCagaagg   1   1   JCK-1
................................................................................ugucgacaagaucCagaaggu   17   1   JCK-3
................................................................................ugucgacaagaucCagaaggu   6   1   JCK-1
................................................................................ugucgacaagaucCagaaggu   11   1   JCK-2
..................................................................................ucgacaagaucuagaaggu   2   0   LT-2
.....................................................................................acaagaucuagaagguacugucgaaua   1   0   LT-1
.....................................................................................acaagaucuagaagguacugucgaaua   1   0   LT-3
...........................................................................................ucuagaagguacugucgaauaag   1   0   LT-3
...........................................................................................ucuagaagguacugucgaauaagaua   1   0   LT-2
...........................................................................................ucuagaagguacugucgaauaagauac   2   0   JCK-1
...........................................................................................ucuagaagguacugucgaauaagauac   1   0   JCK-2
...........................................................................................ucuagaagguacugucgaauaagauac   1   0   JCK-3
............................................................................................cuagaagguacugucgaauaagau   2   0   LT-2
.............................................................................................uagaagguacugucgaaua   1   0   LT-2
..............................................................................................agaagguacugucgaauaagaua   6   0   LCK-3
..............................................................................................agaagguacugucgaauaagaua   1   0   LT-2
...............................................................................................gaagguacugucgaauaaga   2   0   LT-2
................................................................................................aagguacugucgaauaagauac   1   0   LT-2
.................................................................................................agguacugucgaauaagaua   3   0   LCK-3
.................................................................................................agguacugucgaauaagauac   4   0   LCK-3
......................................................................................................................agauacggaggaaggaucaacuuua   1   0   LT-1
........................................................................................................................auacggaggaaggaucaac   1   0   LT-1
.........................................................................................................................uacggaggaaggaucaacu   2   0   LT-3
...............................................................................................................................................................................................uuauuguucguauuuuuuuuc   3   0   LT-2
..............................................................................................................................................................................................................uuuuucccagauuagauuuccc   1   0   LT-3
```
